# Supplementary figures and images for: Adaptive control of movement deceleration during saccades
Source: PLoS Comput Biol. 2021 Jul 6;17(7):e1009176. doi: 10.1371/journal.pcbi.1009176 (PMC8284628; doi:10.1371/journal.pcbi.1009176)

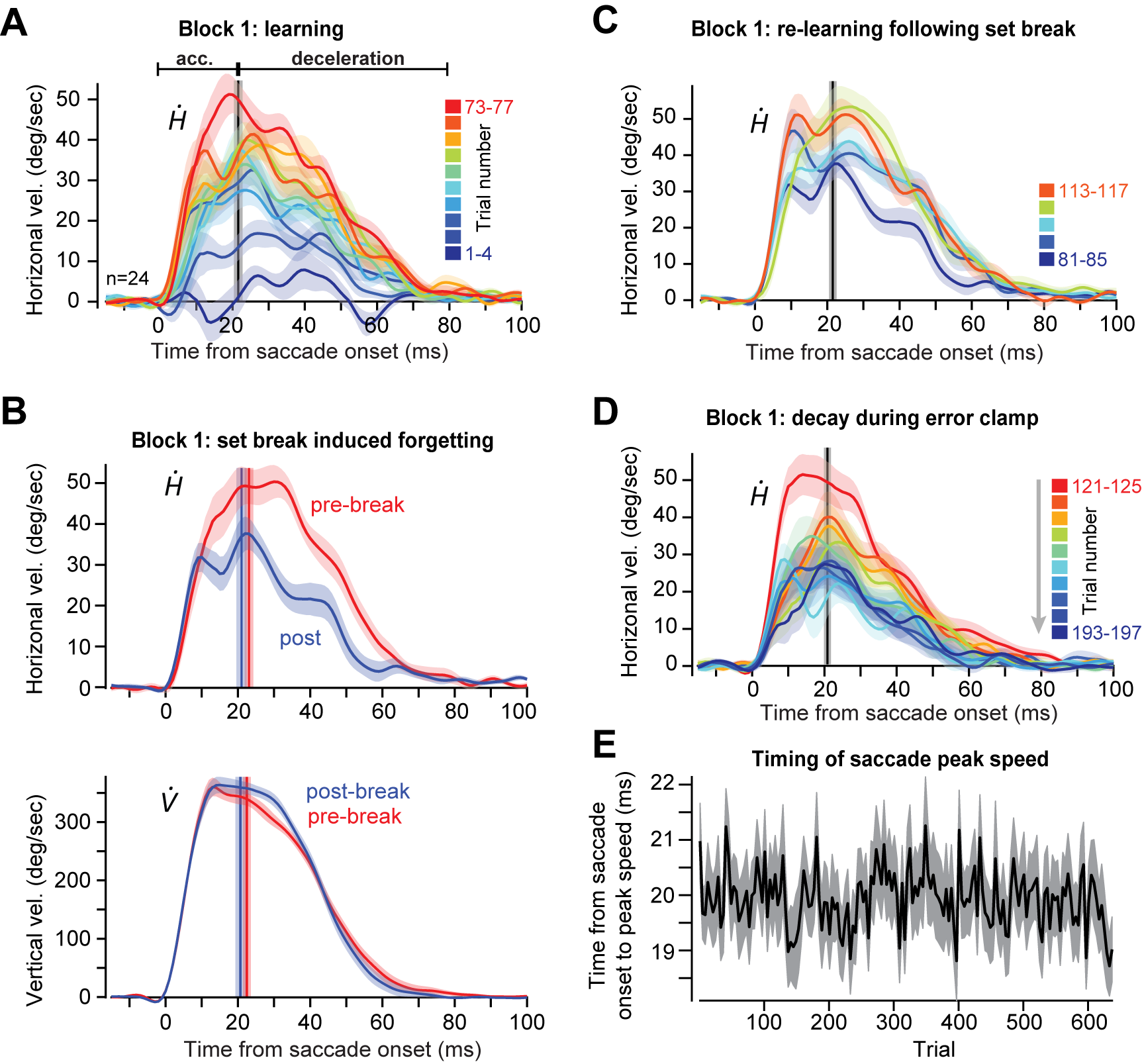

Supplement: S1 Fig — In the main text, we quantified adaptation as the perpendicular displacement of the eye during the acceleration and deceleration phases of the saccade. To calculate this displacement, we integrated horizontal velocity before and after peak speed. Here we show the horizontal velocity during various periods of the first block in Exp. 1. Data are within-subject change in velocity with respect to baseline. Vertical lines indicate timing of peak speed, thus separating the acceleration and deceleration phases of the movement. A. Horizontal velocity during the initial adaptation period. B. Effect of set break on horizontal and vertical velocities. The set break caused decay in horizontal velocity, primarily during the deceleration period (top). The decay was larger in the deceleration period (-0.11 ± 0.33 deg/sec, t(23) = -0.35, p = 0.73 for the acceleration period, -10.08 ± 2.07 deg/sec, t(23) = -4.88, p = 6.33X10-5 for the deceleration period). Vertical velocity did not show decay following a set break (bottom, +7.09 ± 2.32 deg/sec, t(23) = 3.06, p = 0.006). C. Re-learning after the set break during the first perturbation block. D. Decay of horizontal velocity during the first error clamp block. E. Timing of peak speed with respect to saccade onset. The timing that categorizes the movement into acceleration and the deceleration phases was computed by the peak speed, which depends on horizonal as well as vertical velocity. Thus, this timing could change over the learning period as the adaptation component increases, influencing the ratio of the two indexes between the acceleration and deceleration phases. We measured the timing of peak speed with respect to saccade onset in Exp. 1 as well as Exp. 2 and found that there were no significant changes in peak speed timing (Exp 1: F(2,69) = 0.004, p = 0.997; Exp 2: F(3,156) = 0.08, p = 0.97). Combined data across the two experiments are plotted here. Shaded error regions are between subject SEM. (TIF) [file pcbi.1009176.s001.tif]

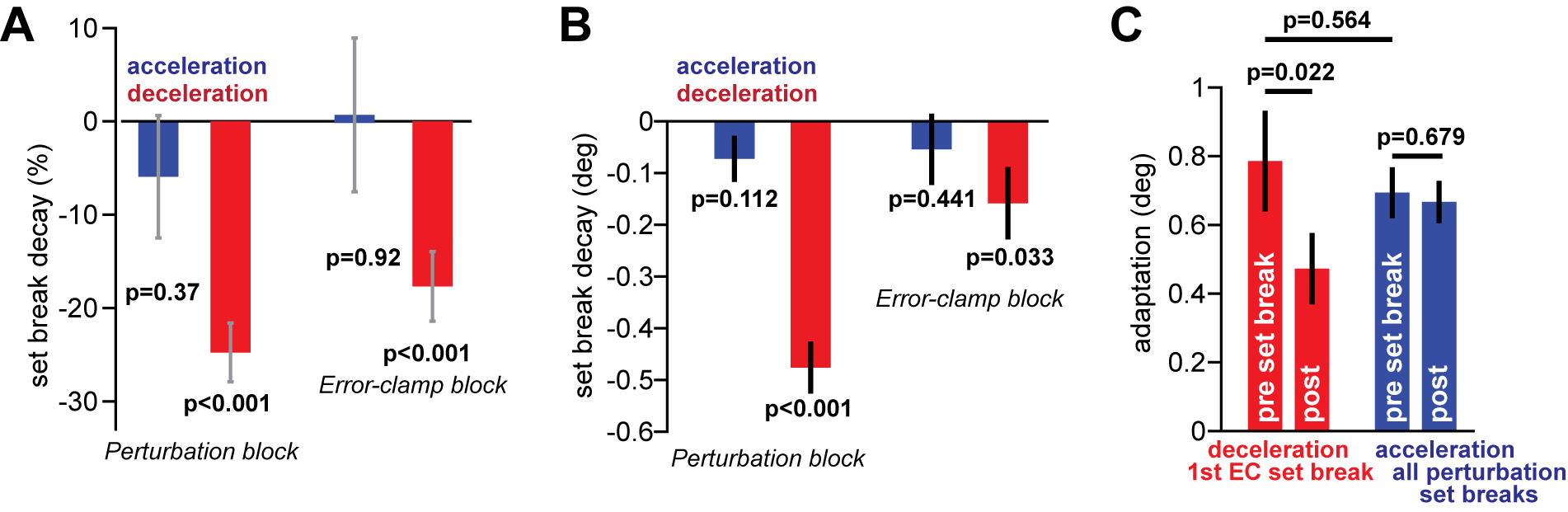

Supplement: S2 Fig — Acceleration period commands generally adapted less than the deceleration period commands. This smaller magnitude could have made it more difficult to find significant changes due to set breaks. To check for this, we performed a series of analyses. A. Percent change in the perturbation blocks (Exp. 1 and 2), and error clamp blocks (Exp. 1). B. Absolute change in the perturbation blocks (Exp. 1 and 2), and error clamp blocks (Exp. 1). C. Here we controlled for differences in the adaptation extent exhibited by acceleration and deceleration period commands. We searched for a period in Exp. 1 where deceleration commands were most similar in magnitude to acceleration commands measured throughout the experiment. The optimal period occurred before the first error-camp period, where deceleration commands reached about 0.8 deg (pre-set break deceleration). Acceleration period commands reached about 0.75 deg throughout the experiment (pre-set break acceleration). Regardless, a decay was present in the deceleration period commands but not acceleration. (TIF) [file pcbi.1009176.s002.tif]

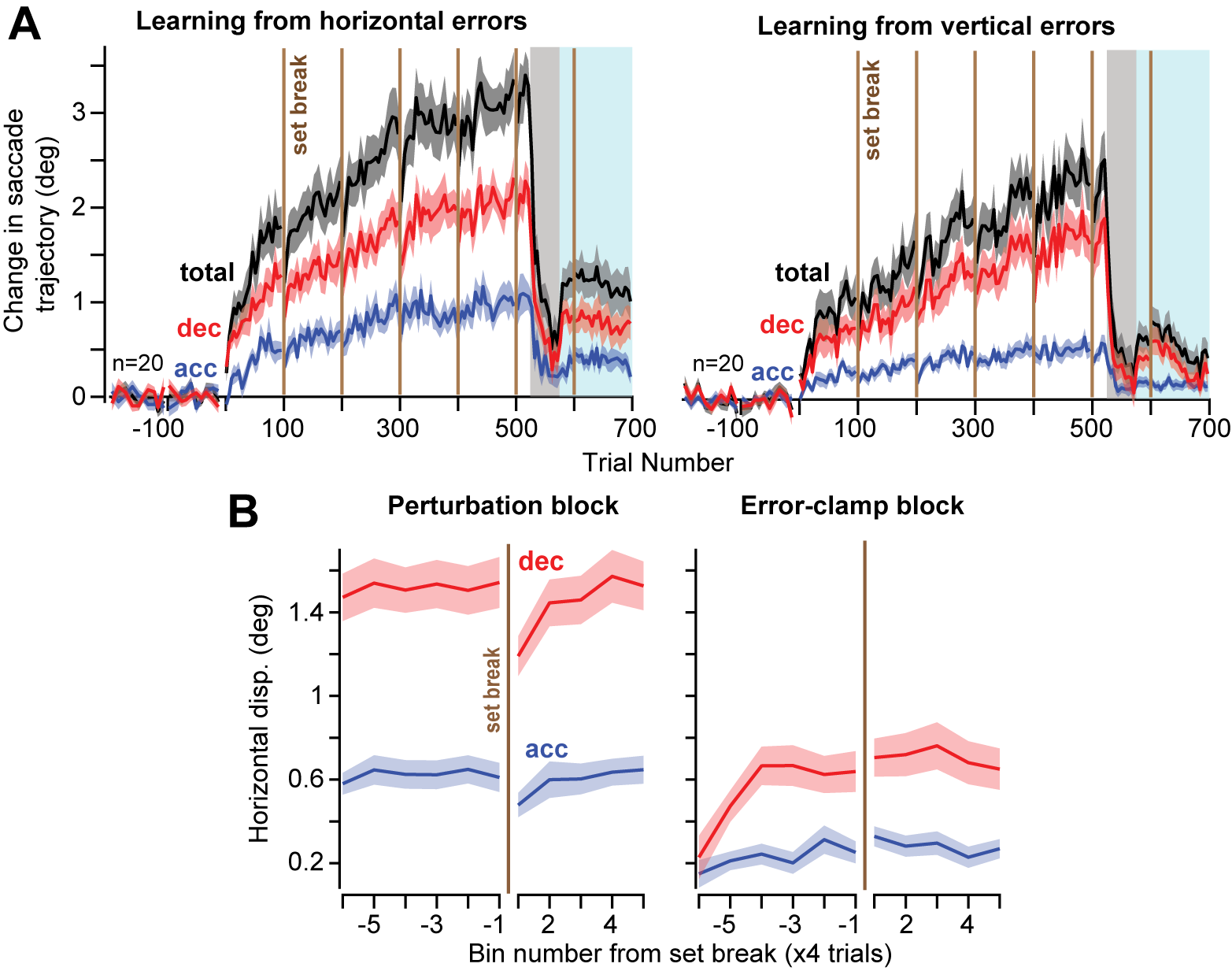

Supplement: S3 Fig — In Experiment 2, an initial perturbation block was followed by extinction and concluded with a block of error clamp trials. Half of the subjects experienced horizontal errors following vertical saccades (left panel), while the other half experienced vertical errors following horizontal saccades (right panel). A. Learning in response to horizontal (left panel) and vertical (right panel) errors. Subjects learned somewhat more from horizontal errors than from vertical, especially during the acceleration period (overall rate of change, 0.005 ± 0.0004 deg/trial vs. 0.002 ± 0.0002 deg/trial, within subject difference t(39) = 7.32, p = 7.89X10-9). B. Effect of set breaks during perturbation and error clamp periods, collapsed across all horizontal error subjects and vertical error subjects. Deceleration period commands decayed more than acceleration period commands during the perturbation period (within subject difference: -0.22 ± 0.07°, t(39) = -3.34, p = 0.001), but neither exhibited decay during the error clamp period, as expected during spontaneous recovery. (TIF) [file pcbi.1009176.s003.tif]

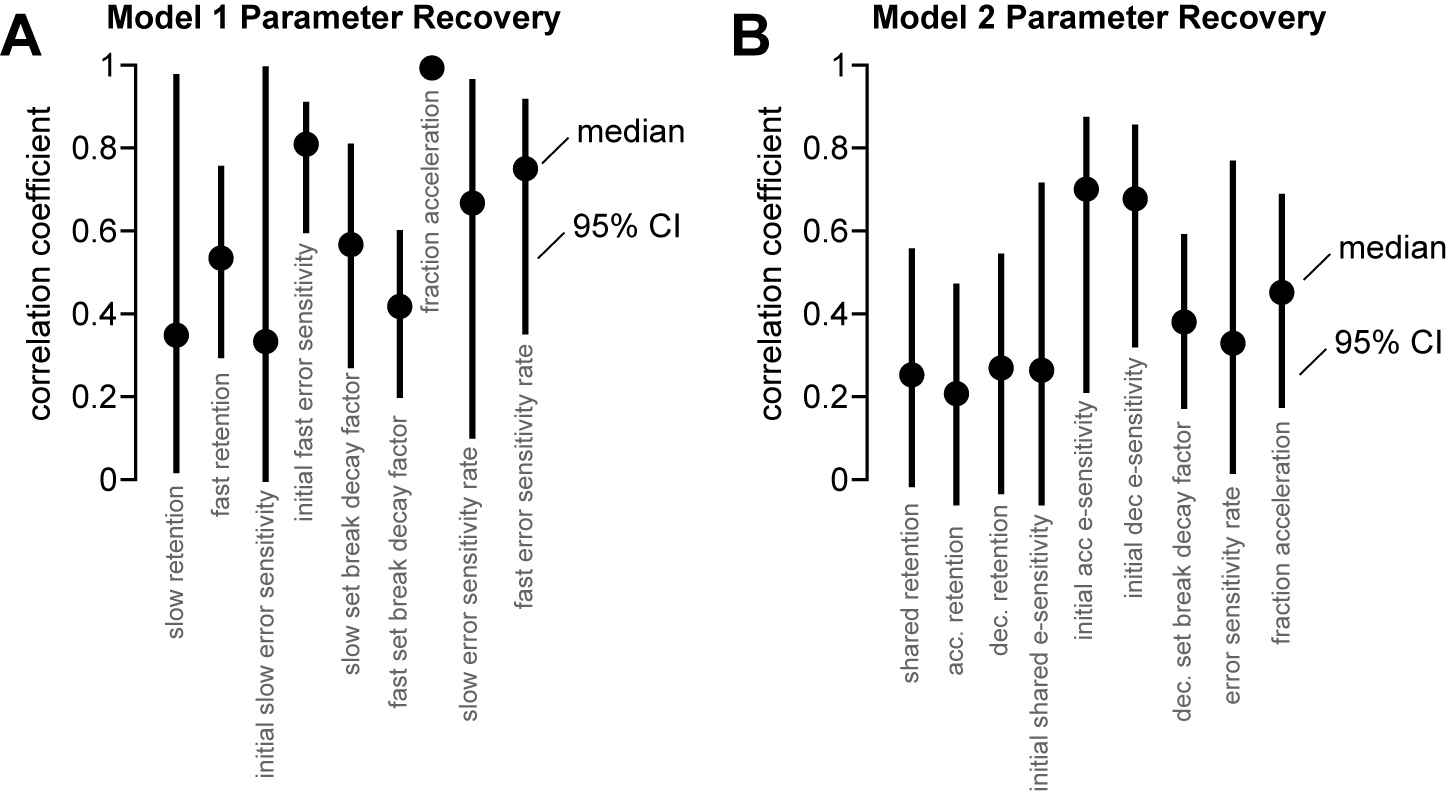

Supplement: S4 Fig — Models 1 and 2 were fit to individual participants in Exp. 1 and Exp. 2, and then used in a parameter recovery analysis. This analysis was conducted via a bootstrapping approach with 1000 batches. In each batch, each parameter set (n = 64 total) was used to simulate noisy behavior with Model 1 (A) or Model 2 (B). Then Models 1 and 2 were fit to the simulated noisy data for each participant. The parameters recovered by each model were compared with the true underlying parameters. To compare the true parameters with the recovered parameters, we calculated Pearson’s correlation coefficient across the 64 participants simulated in a given batch. We repeated this process 1000 times, varying the seed for the random number generator used in each noisy simulation. We then computed the median correlation coefficient, as well as 95% confidence intervals, for each parameter in Models 1 and 2. The higher this correlation, the better the fitting robustness. Black dots represent the median correlation coefficient across 1000 simulated batches, and lines represent 95% confidence intervals. (TIF) [file pcbi.1009176.s004.tif]

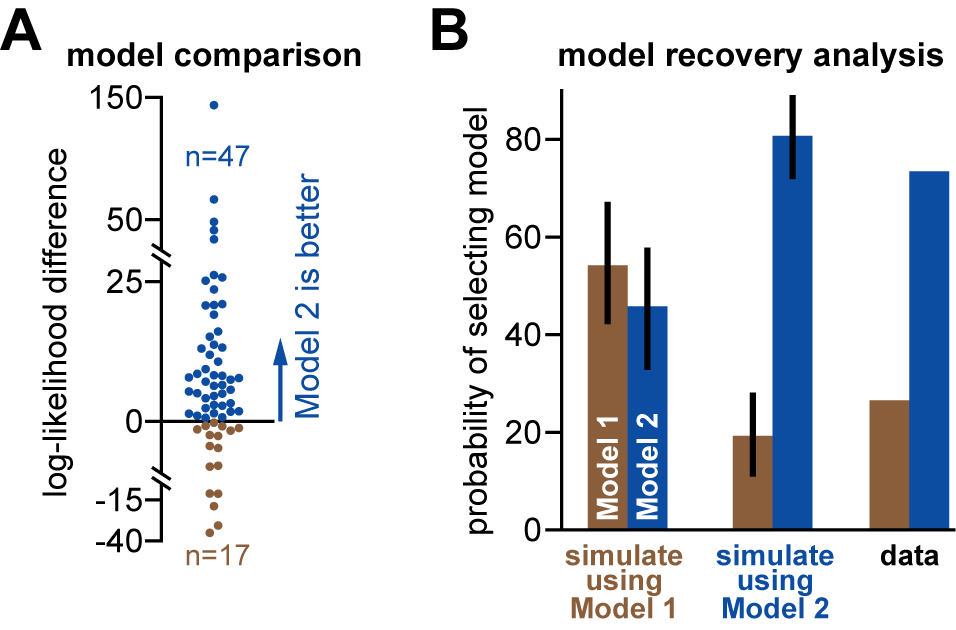

Supplement: S5 Fig — A. We calculated the log-likelihood for observing each participant’s data given Model 1 and Model 2. In panel A we subtract these two likelihoods for each participant: positive values indicate that Model 2 is more likely to explain the participant’s data. B. We performed a model recovery analysis to determine if the log-likelihood was biased toward either Model 1 or 2, given the design of Experiments 1 and 2. We used a simulation to produce data either with Model 1 or 2, and then calculated the log-likelihood for the true model and the opposing model. For each simulation, we calculated the fraction of participants that BIC or AIC selected for a given model. We did this process 1000 times, each time re-simulating behavior for each subject. At left we simulated the experiments using Model 1. In the middle we simulated the experiments using Model 2. At right we show the probability that a participant was better described by Model 1 or Model 2 in the actual data. This model recovery analysis demonstrated that AIC and BIC were both dependable measures that could be used for model comparison; when data were simulated with Model 1, the log-likelihood measure was more likely to recover Model 1. When data were simulated with Model 2, the log-likelihood measure was more likely to recover Model 2. The bars show the mean. Lines indicated 95% confidence intervals across the 1000 simulations batches. (TIF) [file pcbi.1009176.s005.tif]

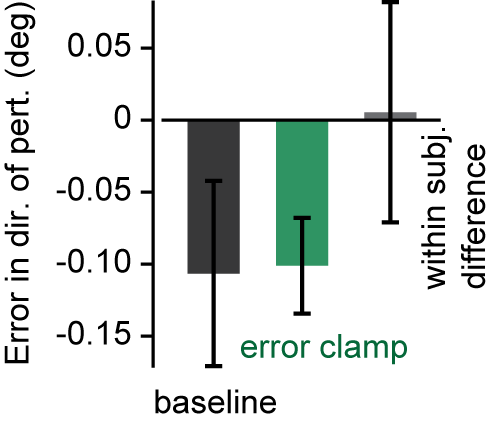

Supplement: S6 Fig — To test whether spontaneous recovery was due to differential decay of multiple learning processes and not to an imperfect implementation of error clamp trials, we calculated the average error in the direction of the original perturbation during the error clamp period of Experiment 2 for each subject and compared it to the average error in the same direction for that same subject during the baseline period (unperturbed trials). We found no difference in error between the two conditions (baseline error: -0.006 ± 0.070 vs. error clamp: -0.10 ± 0.030, within subject difference: -0.10 ± 0.080, t(39) = -1.24, p = 0.22). It should be noted that the visual target occupied a square area of 0.250, and these error measurements are with respect to the center of that area, so the gaze location is still within the bounds of the visual target under both conditions. (TIF) [file pcbi.1009176.s006.tif]

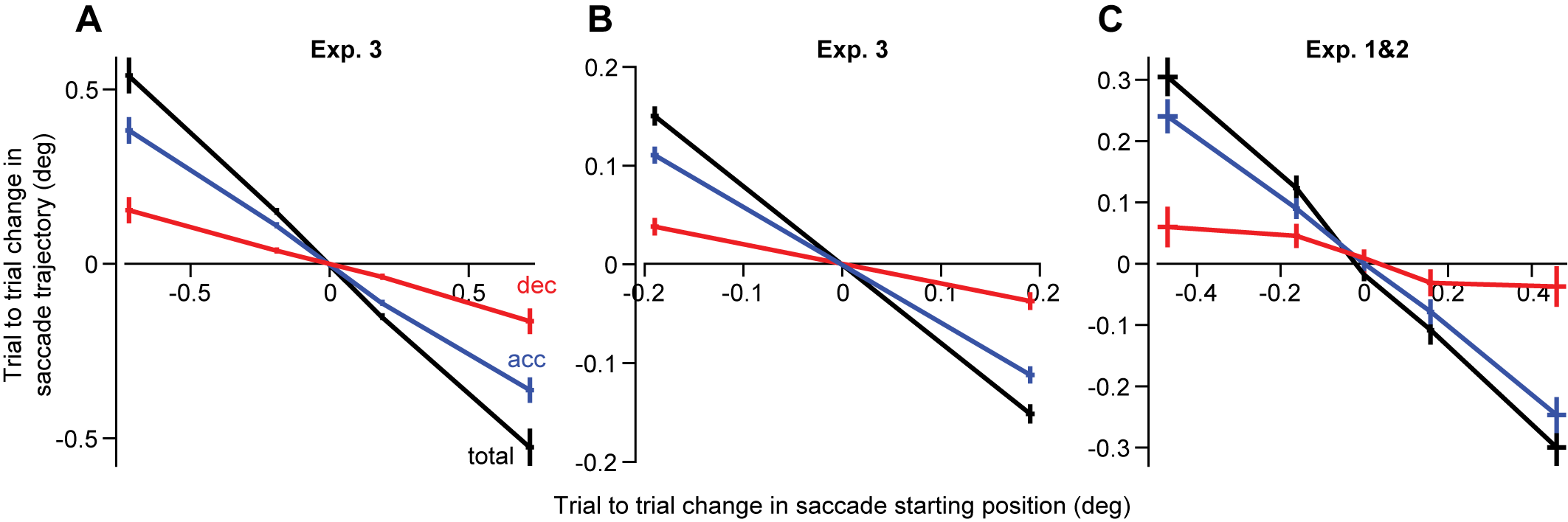

Supplement: S7 Fig — The y-axis quantifies the trial-to-trial change in saccade trajectory during the acceleration and deceleration phases of the saccade. The x-axis shows CHSP of the saccade in trial n with respect to the saccade in the previous trial. A. Effect of change in horizontal starting position. Data from Exp. 3. Leftmost bin is CHSP < -0.5°, next is -0.5° < CHSP < 0°, third bin is 0° < CHSP < 0.5°, and the rightmost bin is CHSP > 0.5°. B. Close up of middle two bins in part A. C. Effect of change in starting position in the baseline periods of Experiments 1 & 2, when there are no errors present (similar to the mean zero perturbation of Exp. 3). Bins are quintiles of CHSP. As in Experiment 3, there is a strong effect that is mainly expressed during acceleration rather than deceleration. This observation adds further support to the idea that there may be separate controllers that contribute to acceleration and deceleration periods of a saccade. (TIF) [file pcbi.1009176.s007.tif]
