# Supplementary material for: Adaptive control of movement deceleration during saccades
Source: PLoS Comput Biol. 2021 Jul 6;17(7):e1009176. doi: 10.1371/journal.pcbi.1009176 (PMC8284628; doi:10.1371/journal.pcbi.1009176)
Supplement: Attachment — Submitted filename: response-to-reviewers-3.docx [file pcbi.1009176.s008.docx]

**Summary of changes to the manuscript:**

- To better compare models, we report both AIC and BIC in the revised manuscript. Both metrics provide support for Model 2 (independent acceleration and deceleration controllers), as compared to Model 1 (coupled controllers), as shown in Supplementary information Fig. S2.
- We updated Models 1 and 2 to better fit the empirical data (the main change was addition of an error sensitivity parameter). We made this change so that the models could reproduce a fundamental aspect of the measured data: savings, i.e., faster and more complete learning with each re-exposure to the perturbation following error-clamp periods and set breaks. Both AIC and BIC suggested that the changes improved the fit: the updated models were more likely to explain the empirical data across Experiments 1 & 2. We also performed further analysis to quantify goodness of fit via parameter recovery analysis (Supplementary information Fig. S3).
- We provide rationale and empirical data as to why it is reasonable to analyze the saccade trajectory based on peak speed.
- Reviewer 2 correctly pointed out an important weakness in our analysis of the set-break data. We corrected this.
- Acceleration period commands exhibited smaller adaptation, which potentially could have affected our ability to measure decay during set breaks. To test for this, we performed additional analysis that considered deceleration period commands during epochs in which the amount of adaptation was similarly small. In this new analysis, we were able to confirm that there was significant decay in the deceleration period commands but not the acceleration period commands, despite similarity in the magnitude of adaptation in the two periods of the saccade. The results are summarized in Supplementary Information Fig. S4
- In the Discussion, we replaced the conceptual model. In the new model the adaptive changes in the acceleration phase were potentially due to changes in the superior colliculus (akin to remapping of the target), whereas the adaptive changes in the deceleration phase were potentially due to contributions from the cerebellum (steering the saccade).

**Reviewer #1**

This paper proposes a novel idea that even a single continuous movement might be generated from multiple controllers with different dynamic properties for adaptation. The data of experiment 3 clearly show that the first and the second halves of the adaptive system has different learning properties. The model-based analysis with the state-space representation of the memory update revealed that two adaptive system components are composed of the fast and the slow memories. Although the current version of the manuscript is written very well and the methods and the results are reasonable, there is a couple of points that should be addressed before it is accepted.

Thank you for reading our manuscript and for your insightful comments.

Major questions.

**Point 1.** 1) In general, how the hypothesis was derived should be written in the Introduction section. In the third paragraph, the authors stated that the timescale of learning was different among phases of a single movement by citing the previously published paper. However, it is unclear why the acceleration and the deceleration phases should separate the single movement and why separating the movement by time was not adopted.
In our earlier work (Chen-Harris et al. 2008), we reported that adaptation in response to cross-axis endpoint errors produced saccade trajectories that exhibited interesting properties: the motor commands that initiated the saccade changed slowly in the direction that would reduce the endpoint error, but late in the same saccade, the motor commands appeared to steer the eyes further. Our analysis in Chen-Harris et al. (2008) relied on division of the saccade trajectory (i.e., its path) into 4 equal length segments. The segments were defined by dividing the length of the saccade into 4 parts along its primary axis (thus, resulting in 4 chords), as shown in Fig. R1A below. In control trials (before adaptation), we measured the slope of each chord in response to targets at (+15,0), (+15,+1), (+15,+2), and (+15,+3) degrees. We measured the chord slopes in these control trials, as shown in Fig. R1B. We then performed the adaptation experiment and presented the target at (+15,0) and again measured the chord slopes. The results are shown in Fig. R1C below.

**Fig. R1.** Analysis of saccade path during cross-axis adaptation. Saccades were made to horizontal targets at 15^o^. To induce adaptation, the target was jumped by 5^o^ along the vertical direction. (A) Each saccade was divided into 4 segments based on the horizontal position. Slope of each segment was measured and labeled as S1, S2, S3, or S4. (B) Control trials in which subjects made oblique saccades to targets at 1, 2, or 3 deg vertical amplitude and 15 deg horizontal amplitude. (C) Data during early, middle, or late trials of adaptation. Note that chord slopes within a saccade increase nonlinearly, indicating presence of curvature. Data from Chen-Harris (2008).

We found that with adaptation, the chord slopes exhibited a remarkable pattern: while the slope gradually increased as the movement progressed toward its endpoint, the rate of change was not linear. Rather, whereas chords 1 and 2 had roughly equal slopes, chord 4 had twice the slope of chords 1 and 2. This illustrated that for the adapted saccades, most of the change was due to commands that arrived late in the movement. Notably, a critical time point during a saccade was roughly at the end of chord 2, that is, midway into the movement. This point along the saccade’s trajectory corresponds approximately to peak speed of the saccade. Thus, empirical evidence from Chen-Harris et al. (2008) provided us with a rationale to divide the saccade based on timing of peak speed, leading to acceleration and deceleration phases.

However, as you noted, by itself this is not a strong piece of evidence. Thus, part of the impetus for Exp. 3 was to ask whether there was independent evidence for the idea that control of the deceleration period is specifically under an adaptive process that is different than the deceleration period. In Exp. 3, we focused on single trial learning, thus allowing us to measure trial-to-trial change in saccade trajectory in response to a single endpoint error (Fig. R2A). We found that following experience of a single error, on the next trial the motor commands changed. We quantified this change via the horizontal and vertical components of the saccade velocity and found that horizontal velocity (Hdot) showed a bimodal response that crossed zero almost precisely at saccade peak speed (Fig. R2B). That is, the motor commands that steered the eyes in the correct direction did not arrive until the deceleration period of the saccade. These results indicated that on average (i.e., across the population), onset of saccade deceleration was a critical timepoint for analysis of Hdot.

**Fig. R2**. Following experience of a single error, correction on the subsequent saccade was primarily during the deceleration period. **(A)** Subjects were presented with a vertical target at 15 deg and occasionally experienced a horizontal error of +5 or -5 degrees. **(B)** Trial-to-trial change in horizontal velocity following experience of a positive or negative endpoint error. Note that the response as measured via Hdot is bimodal, crossing the zero axis almost precisely at onset of deceleration. **(C)** Onset time of correction in the horizontal velocity following experience of a horizontal endpoint error. Mean onset time is about 3 ms following start of the deceleration period. **(D)** Data from Experiments 1 & 2, measuring the response to error following experience of error on the very first trial (velocity on trial 2 minus trial 1, in the direction of the perturbation). The component of Hdot that corrects for the error experienced in the previous trial is entirely in the saccade deceleration period.

Next, we searched for the time during a saccade’s trajectory in which Hdot indicated onset of the corrective command. That analysis showed that the onset of Hdot was statistically indistinguishable from time of saccade peak speed (Fig. R2C). To confirm this finding, we considered the data across all our 100+ subjects and measured how the saccade velocity changed from one trial to the next in response to a single experience of an endpoint error. The results clearly illustrated that the adapted response was present only in the deceleration period of the saccade (Fig. R2D).

In summary, our experiments in Chen-Harris et al. (2008) demonstrated that while adapted motor commands were present during the entire trajectory of the saccade, they were not expressed equally during the trajectory: a nonlinearity appeared to take place at midpoint along the saccade path, which roughly corresponds to saccade peak speed. This empirical evidence provided us with a reason to analyze data based on division of the movement into acceleration and deceleration periods. However, to independently test the idea, we performed Exp. 3 and found that the adaptive response to a single error occurred only during the deceleration period. These empirical lines of evidence compelled us to analyze the data using the time point when the peak speed occurred. These ideas are now highlighted in the both the Introduction and the Results sections.

**Point 2.** Major 2. The timing that categorizes the movement by the acceleration and the deceleration phases was computed by the peak speed, sqrt(H’^2+V’^2). Thus, this timing should be changed over the learning period as the adaptation component H’ increases, which influences the ratio of the two indexes between the acceleration and deceleration phases. Thus, the estimated relative contribution of the deceleration phase increases over the trial as the timing of the peak speed shifts. If this is the case, the different learning speed shown in figure 1E is the artifact of this timing shift. To refute this possibility, the authors should plot how the timing of peak speed changes over trials.

We measured the timing of peak speed with respect to saccade onset in Exp. 1 as well as Exp. 2 and found that there were no significant changes in peak speed timing in both experiments (Exp 1: F(2,69)=0.004, p=0.997; Exp 2: F(3,156)=0.08, p=0.97). The data are plotted in the figure below. Thus, the timing of peak speed appeared to remain fairly constant throughout the experiment.

|  | **Fig. R3.** Timing of peak speed with respect to saccade onset. Data combined across experiments 1 and 2. Bin size is 4 trials. |
| --- | --- |

**Point 3.** Major 3. Model comparison based on BIC is solid. However, in general, relying on only one index is risky. As was done in many papers, reporting BF, AIC in addition to BIC is recommended.
Thank you for this important suggestion. To better compare Models 1 and 2, we now report both AIC and BIC in the revised manuscript. In response to comments of reviewers 2 and 3, we first changed the models so to allow for variability in error sensitivity. In the response below, we first document that the updated models were better fit to the empirical data than the older models (Fig. R3). We then compare Model 1 with Model 2, and find that Model 2 is consistently a better match to the data (Fig. R4).

In response to questions raised by Reviewers 2 & 3 (Point 12, below), we updated Models 1 and 2. The main change was addition of an error sensitivity parameter (Herzfeld et al., 2014). We made these changes so that each model could exhibit savings, i.e., faster and more complete learning with each re-exposure to the perturbation following error-clamp periods and set breaks. As a result, the updated models each had two additional parameters than their predecessors. Both AIC and BIC suggested that the change to the models were justified: the variable error sensitivity models were more likely to explain the measured data across Experiments 1 and 2.

Results for Model 1 are shown in Fig. R4**A** below. AIC suggested that 46 participants were better described by the variable error sensitivity model, whereas 18 were better described by the constant error sensitivity model (an average AIC improvement of 24 across individual participants, t(63)=6.05, p<0.001). Summed AIC evidence across all participants favored the variable error sensitivity model (total AIC difference of 1536). Similar results were found with BIC.

Results for Model 2 are shown in Fig. R4**B** below. AIC suggested that 46 participants were better described by the variable error sensitivity model, whereas 18 were better described by the constant error sensitivity model (an average AIC improvement of 26.19 across individual participants, t(63)=5.61, p<0.001). Summed AIC evidence across all participants favored the variable error sensitivity model (total AIC difference of 1676). Similar results were found with BIC.

In summary, in the revised manuscript, the original constant error sensitivity models were replaced by the variable error sensitivity models. AIC and BIC suggested that the revised models were better fit to the measured data.

**Fig. R4**. Comparison of the current version of the models (variable error sensitivity) with the original version in the initial submission of the manuscript.

Next, we compared the two models and found that Model 2 was a better fit to the data than Model 1 (Fig. R5). AIC suggested that 45 participants were better described by Model 2, whereas 19 participants were better described by Model 1 (an average AIC improvement of 18.4 across individual participants, t(63)=3.14, p=0.003). Summed AIC evidence across all participants favored Model 2 (total AIC difference of 1175). BIC provided similar results.

**Fig. R5**. Comparison of Model 2 with Model 1.

In summary, the updated model comparisons provide even stronger evidence in favor of independent acceleration and deceleration controllers. We thank the reviewer for their suggestion.

**Point 4.** Minor 1. I request additional references to the saccadic adaptation task in line 85.

This paragraph attempts to provide a rationale for Exp. 2. The results of Exp. 1 suggested that the slow and fast adaptive processes may individually shape different parts of a single movement: fast process expressed during the acceleration phase, slow process expressed during the deceleration phase. That model predicted how the states should evolve during the period of spontaneous recovery. This led us to Exp. 2 in which we measured saccade trajectories during spontaneous recovery and found that the empirical data was inconsistent with the model predictions. We re-wrote this paragraph to better explain this thought process.

**Point 5.** Minor 2, L129. How long is the duration of the set-breaks?

As noted in Methods, set-breaks are 30 seconds in duration. We have also added a parenthetical note mentioning this duration to the first mention of the error clamp trials in the results section.

**Point 6.** Minor 3, L177. It took me a while to notice that (0,15) the target's position at (horizontal, vertical) in degrees. Just a brief note about this notation is helpful to read.

Done.

**Point 7.** Minor 4, L717. The definition of the error is reasonable in terms of the state-space model of memory update. It is helpful to understand if the authors briefly describe how the brain observes the error in the saccadic adaptation task. Is it via a corrective saccade or the retinal slip of the target stimuli?

Errors that lead to adaptation appear to be due to unexpected post-saccadic activity among the neurons in the superior colliculus. This inference comes from the observation that during the period following completion of a saccade, low-intensity stimulation of the colliculus results in production of complex spikes in the cerebellum, and trial-to-trial behavioral changes that resemble saccade adaptation. The representation of error is in retinal coordinates. We added the following to this section.

“In biological terms, the error is a vector that reflects position of the target with respect to the fovea in the post-saccadic period (Shadmehr 2020). This is inferred by the fact that low-intensity stimulation of the superior colliculus at around 80 ms following completion of a saccade results in production of complex spikes in the cerebellum (Soetedjo et al., 2019), and is followed by behavioral changes that resemble saccade adaptation (Kaku et al., 2009; Soetedjo et al., 2009). Here, the perturbation was perpendicular to the direction of the main target, and thus we represented error as a scalar quantity: the distance between saccade endpoint and target position following saccade end.”

**Point 8.** Minor 5, L800-L803. Why is using the individual commands rather than the total commands reasonable? How were these two costs examined before one was selected?

We agree that we could have motivated this cost function more clearly in the text. Generally, the state-space adaptation model is used to track how a scalar variable (e.g., reach direction, saccade amplitude, etc.) changes in response to error. Models 1 and 2, however, are concerned with two variables, the commands issued during acceleration and deceleration periods. In other words, rather than model the overall saccade amplitude, we model two individual movement period components. The cost function we use informs the mathematical model of each component – otherwise, the model is unaware how much acceleration and deceleration learning occurred. For example, consider Model 1: a key parameter here is *p*, the proportion of adaptation expressed during the acceleration period. If the model is not fit with knowledge of acceleration and deceleration commands, it has no ability to specify a value for *p*. In other words, Model 1 supposes that acceleration and deceleration commands are related by a scaling factor; there is no way to specify this scaling factor without specifying the individual acceleration and deceleration period commands within the cost function.

In the case of Model 2: total saccade amplitude depends on 4 individual states, a slow state and fast state for each movement period. Without using the cost function to “force” certain states to apply to one period or the other, there is no direct mapping between a given state and the movement period it serves.

**Point 9.** Minor 6, L849. How was the robustness of fitting examined?

Thank you for noting this important detail. In the original manuscript, we did not report any particular measures regarding the robustness in our fitting technique. In Fig. 5, we fit mean behavior across all participants and across Experiments 1 and 2 simultaneously. This technique is expected to improve robustness in identifying the underlying parameters due to a reduction in noise (by averaging across participants) and preventing each model from overfitting data from one experiment (by fitting both experiments at the same time, assuming a common parameter set can explain both data sets). Overall, we would expect either technique to experience robustness similar to fitting two-state models of adaptation (see Albert and Shadmehr, 2018). This is because Model 1 is a two-state model constrained to fit acceleration and deceleration period behavior. Model 2 is a four-state model, but two states are confined to the acceleration period and two states are confined to the deceleration period, and so effectively this is essentially a model composed of two parallel two-state models.

Nevertheless, in light of your comment, we do feel that a more direct examination of fitting robustness is warranted. Thus, in the revised manuscript, we explored fitting robustness using the following method. We simulated behavior for each of the 64 participants a total of 1000 times using Models 1 and 2. For each batch (of 64 subjects) we calculated the correlation coefficient between the true underlying parameters and the parameters recovered by the model fit. The higher this correlation, the better the fitting robustness. Below, we show the 95% CIs, and median value describing this correlation coefficient for each parameter value. Robustness appeared to be high: our ability to recovery Model 1 parameters exhibited a median correlation ranging between 0.33 and 0.99 (0.60 ± 0.22, mean ± SD). Our ability recovery Model 2 parameters exhibited a median correlation ranging between 0.52 and 0.99 (0.75 ± 0.16, mean ± SD).

**Fig. R6**. Parameter recovery analysis.

Thus, we feel that this parameter recovery analysis suggests that our method for identifying model parameters was robust. We highlight this analysis in the revised paper.

**Reviewer #2**

This manuscript examines saccade adaptation from a behavioral and modeling perspective. Consistent with their prior findings, the authors note that during cross-axis adaptation, saccade kinematics change in a manner inconsistent with typical oblique saccades, having a much larger corrective component at the end of the movement that is more responsive to errors; additionally, both the early and late components exhibited spontaneous recovery. The authors noted that the popular two-state model is unable to capture these temporal dynamics, and instead propose an alternative model in which the early and late phases of the saccade are governed by separate controllers.

Thank you kindly for taking the time to review our work.

**Point 10.** There are 3 major issues in this manuscript. The first is that even under the best of circumstances (e.g., non-adapted saccades), the horizontal and vertical components of oblique saccades are not obligately coupled (e.g., King et al J Neurophysiol 1986, Becker and Jurgens Vis Res 1989, etc). Specifically, the two components of the saccade do not necessarily start and stop at the same time, their peak velocities do not align well, and the shorter component is typically stretched, possibly asymmetrically. Practically speaking, this poses a challenge when attempting to examine the acceleration and deceleration phases of an oblique saccade – particularly one generated via adaptation; it seems highly unlikely that the peak speed (considering the horizontal and vertical components together) is an appropriate place to divide the individual components into acceleration and deceleration phases. If a given component is not appropriately divided, this in turn could lead to misleading conclusions about the extent to which changes are occurring in the early and late phases of that component (e.g., if not enough of the true acceleration phase is labeled as such it could explain why changes are really only observed in the deceleration phase). This calls the main behavioral findings of this manuscript into question.

As you noted, King et al. (1986) closely examined kinematics of oblique saccades and noted that despite the fact that premotor and motoneuronal pools associated with vertical and horizontal saccades are largely separate, during oblique saccades activities in these neurons are likely coordinated, possibly by a common source that may be the superior colliculus. Becker and Jurgens (1990) described an alternative model in which this coordination was via real-time internal feedback, possibly a state-estimator. In our own work, the coordination of horizontal and vertical components of saccades is readily visualized in the effect of reward on saccade vigor: when the value of the stimulus is increased, saccade peak velocity increases. This increase is present in both the horizontal and vertical components of oblique saccades (Shadmehr and Ahmed, 2020). Your comments made us think about future experiments in which we can use vigor modulation of oblique saccades to quantify the precise coordination in the two components. For example, such future experiments might be able to test whether an increase in stimulus value increases speed of the vertical and horizontal components of an oblique saccade equally.

Regardless, you raise an important question: is it appropriate to analyze cross-axis adaptation data using peak saccade speed as a critical time marker? Here, we provide the empirical data that suggested to us that the transition from acceleration to deceleration may be a particularly salient period during the saccade to measure the adaptive response to endpoint errors.

In our experiments, saccades were made to visual targets that were always along the cardinal directions: horizontal or vertical. That is, the visual cue was always horizontal or vertical. However, with adaptation, the brain learned to alter the motor commands in response to the visual targets, thus producing slightly oblique saccades. Our concern was to quantify the changes in the trajectory of the saccade during adaptation.

In an earlier work (Chen-Harris et al. 2008), we reported that adaptation in response to cross-axis endpoint errors produced saccade trajectories that exhibited interesting properties: the motor commands that initiated the saccade changed slowly in the direction that would reduce the endpoint error, but late in the same trajectory, the motor commands appeared to aggressively steer the eyes further in the same direction. That analysis relied on division of the saccade trajectory (i.e., its path) into 4 segments. The segments were defined by dividing the length of the saccade into 4 parts along its primary axis, thus resulting in 4 chords, as shown in Fig. R1A below. In control trials, we measured the slope of each chord in response to targets at (+15,0), (+15,+1), (+15,+2), and (+15,+3) degrees. We measured the chord slopes in these control trials, as shown in Fig. R1B. We then performed the adaptation experiment and presented the target at (+15,0) and again measured the chord slopes. The results are shown in Fig. R1C below.

**Fig. R1.** Analysis of saccade path during cross-axis adaptation. Saccades were made to horizontal targets at 15^o^. To induce adaptation, the target was jumped by 5^o^ along the vertical direction. (A) Each saccade was divided into 4 segments based on the horizontal position. Slope of each segment was measured and labeled as S1, S2, S3, or S4. (B) Control trials in which subjects made oblique saccades to targets at 1, 2, or 3 deg vertical amplitude and 15 deg horizontal amplitude. (C) Data during early, middle, or late trials of adaptation. Note that chord slopes within a saccade increase nonlinearly, indicating presence of curvature. Data from Chen-Harris (2008).

We found that with adaptation, the chord slopes exhibited a remarkable pattern: while the slope gradually increased as the movement progressed toward its endpoint, the rate of change was not linear. Rather, whereas chords 1 and 2 had roughly equal slopes, chord 4 had twice the slope of chords 1 and 2. This illustrated that for the adapted saccades, most of the change was due to commands that arrived late in the movement. From these data we inferred that for an adapted saccade, a critical point was roughly at the end of chord 2, that is, midway into the movement. This spatial location along the saccade’s trajectory corresponds approximately to when the saccade acquires its peak speed.

A second line of evidence regarding importance of peak speed was uncovered in the current paper. In the current paper, subjects were presented with a target at 15 deg along the vertical direction and then experienced an error in the horizontal direction. In Exp. 3, these errors were random, thus allowing us to measure change in saccade trajectory following experience of a single endpoint error, as shown in Fig. R2A. We found that following experience of an error, on the next trial the motor commands changed. We quantified this change via the horizontal and vertical components of the saccade velocity and found that horizontal velocity (Hdot) showed a bimodal response that crossed zero almost precisely at saccade peak speed (Fig. R2B). That is, the motor commands that steered the eyes in the correct direction did not arrive until the deceleration period of the saccade. In summary, these results indicated that on average (i.e., across the population), onset of saccade deceleration was a critical timepoint for analysis of the adaptive response to error.

**Fig. R2**. Following experience of a single error, correction on the subsequent saccade was primarily during the deceleration period. **(A)** Subjects were presented with a vertical target at 15 deg and occasionally experienced a horizontal error of +5 or -5 degrees. **(B)** Trial-to-trial change in horizontal velocity following experience of a positive or negative endpoint error. Note that the response as measured via Hdot is bimodal, crossing the zero axis almost precisely at onset of deceleration. **(C)** Onset time of correction in the horizontal velocity following experience of a horizontal endpoint error. Mean onset time is about 3 ms following start of the deceleration period. **(D)** Data from Experiments 1 & 2, measuring the response to error following experience of error on the very first trial (velocity on trial 2 minus trial 1, in the direction of the perturbation). The component of Hdot that corrects for the error experienced in the previous trial is entirely in the saccade deceleration period.

However, we wanted to know whether on a single subject basis the reliance on peak speed was valid. Thus, in Exp. 3, for each subject we searched for the time in which Hdot indicated onset of the corrective command (Fig. R2C). That analysis showed that the onset of Hdot was statistically indistinguishable from time of saccade peak speed. To confirm this finding, we considered the data across all our 100+ subjects and measured how the saccade velocity changed from one trial to the next in response to a single experience of an endpoint error. The results clearly illustrated that the adapted response was present only in the deceleration period of the saccade (Fig. R2D).

In summary, our experiments in Chen-Harris et al. (2008) illustrated that while adapted motor commands were present during the entire trajectory of the saccade, they were not expressed equally during the trajectory: a nonlinearity appeared to take place at midpoint along the saccade path, which roughly corresponds to saccade peak speed. We found an independent confirmation of this conjecture in Exp. 3 of the current manuscript, in which we measured the single trial response to error and observed that Hdot changed sign and corrected the saccade almost precisely when the saccade began its deceleration phase. These empirical lines of evidence compelled us to analyze the data using the time point when the peak speed occurred.

**Point 11.** The second issue is that is already well known that the early and late phases of a saccade are critically distinct in how they are controlled: early in the movement the motor command primarily driven by a preplanned command, whereas late in the movement the motor command is influenced by corrections (e.g., based on a forward-model). Even if we ignored the issue above, differences in online corrections could potentially explain why different phases of the movement adapt to different extents; for example, it may have to do with whether adaptation drives changes in the forward model or the controller, rather than needing to invoke a model in which the early and late phases of a movement are driven by separate controllers. Indeed, the authors’ previous work regarding fatigue (e.g., Xu-Wilson 2008) suggests that the ability to generate late corrections is critical for maintaining saccades accuracy even in non-adapted saccades, and thus it is not surprising that the deceleration phase responds sooner and to a greater extent in response to observed errors. The authors thus need to consider the presence of late-movement motor corrections when attempting to explain their findings (e.g., lines 268-275).

It is reasonable to assume that early in the saccade, motor commands are largely responding to the visual stimulus, likely driven by activity in the superior colliculus. However, late in the same movement the motor commands are influenced by a correction, possibly from the cerebellum. Mathematically, this assumption can be described via a scheme in which a controller specifies the motor commands, whereas the forward model makes predictions that depend on those commands, thus allowing the controller to correct the saccade further as it progresses in time.

We formally modelled this process in Chen-Harris et al. (2008) in the context of cross-axis adaptation (Fig. R3A, below). That model consisted of an optimal controller and a forward model. The results illustrated four possibilities.

(1) In response to error, the brain learns to remap the target. In this case, presentation of the visual cue at (0,15) results in a remapping that aims the saccade to an endpoint other than the visual target. This produces a change in the saccade goal. The consequence is a roughly straight saccade to the remapped location. The prediction is that if this was the only mechanism of learning, then saccades made to oblique targets in control trials and adapted saccades would not differ from each other. As shown in the current paper, this possibility is inconsistent with our empirical data.
(2) In response to error, the forward model adapts. In this case, saccade trajectory will be curved. This appears consistent with our empirical data in both Chen-Harris et al. (2008) and the current paper. This is Model 1 of the current paper. However, as we note below, the differing rates of decay during acceleration and deceleration periods of the saccade appear inconsistent with this model. This idea, and its limitations, are described in more detail below.
(3) In response to error, the controller adapts. Adaptation of the controller is modeled through equations that describe dynamics of the eye and its sensory consequences. However, because the forward model has not adapted, eye trajectory is corrected in the “wrong” direction. This prediction is inconsistent with our empirical data.
(4) Both the controller and the forward model adapt. In this case, in the fully adapted system saccade trajectory is similar to the control condition in which an oblique target is presented. Complete adaptation of both the controller and the forward model are inconsistent with our data. However, partial adaptation in each system may be consistent with the results obtained in the current manuscript.

**Fig. R3**. Modeling saccade adaptation in the cross-axis paradigm via change in the controller, forward model, or both. (A) Schematic for the model. The teaching signal that guides learning is the retinal error at end of saccade. In principle, this teaching signal can result in: remapping of the target (forward model of the target), change in the forward model of the oculomotor plant, or change in the optimal controller, or a change in the controller and the forward model. (B) Adaptation paradigm. (C) Results of simulations. If learning is via a change in a forward model of the target, then effectively the system remaps the visual stimulus to a new location. Saccades kinematics in adaptation trials would remain similar to control, oblique trials. (B) Forward model of the oculomotor plant adapts, but not the controller. Adapted saccades become curved. (C) The controller adapts, but not the forward model. Adapted saccades show concave curvature. (D) Both the controller and the forward model adapt. If the adaptation is complete, then saccades will be similar to control, oblique saccades. From Chen-Harris et al. (2008).

Results of Chen-Harris et al. (2008) led us to suggest that the most likely scenario was one in which the forward model underwent adaptation. While chord 1 changed slowly with respect to error, during set breaks chord 1 exhibited little or no forgetting (Fig. R4). In sharp contrast, chord 4 showed substantial learning from error, but also substantial forgetting (Fig. R4). Thus, results of Chen-Harris et al. (2008) led us to conjecture the forward model was the basis of adaptation, and this adaptation was dominated by a two-state process: one that learned slowly and showed little decay during set breaks, and another that learned more but was susceptible to decay during set breaks.

These results led us to the experiments in the current manuscript. In Exp. 1, we reproduced the findings of Chen-Harris et al. (2008). In Exp. 2 we measured the time course of change in motor commands during a spontaneous recovery paradigm, and found that both components of the adapted response showed spontaneous recovery. All in all, the data suggested that each movement phase was supported by two states of learning, and that these states were computed by two distinct control systems which were best highlighted in their response to a single error (Experiment 3) or after the occurrence of a set break (Experiments 1 and 2).

|  | **Fig. R4**. Multiple timescales of change in various components of saccade trajectory. The plot displays chord 1 and chord 4 slope of saccades during cross-axis adaptation (see definition in Fig. R1). Each training set is about 100 trials in length. Note the set-break effect on S4. Data from Chen-Harris et al. (2008). |
| --- | --- |

In the context of the model in Fig. R3, the superior colliculus represents the optimal controller, whereas the cerebellum represents the forward model. If both the controller and the forward model adapt, then adaptation should produce a change in both the activity of the colliculus, and the activity of the cerebellum. While no data is available regarding this in the cross-axis paradigm, in other forms of saccade adaptation activity in the superior colliculus **does not** appear to show a change during saccade adaptation (Frens and Van Opstal 1997; Quessy et al. 2010). In contrast, there is a large body of data pointing to the cerebellum as an important system that contributes to changes in motor commands and guides adaptation. We point out this view and suggest it as a distinct possibility in the Discussion.

Importantly, our experimental data allow us to consider the possibility of whether adaptation is solely due to changes in the forward model. If this were the case, then the model reduces to that shown in Fig. 5A in the main manuscript (Model 1): a single process with two states that contribute to learning, with a fraction of the adaptation expressed during acceleration, and another fraction expressed during deceleration. Indeed, we found that Model 1 was able to reproduce many features of the empirical data. For example, the deceleration period commands adapted more rapidly than the acceleration period commands. This was because in Model 1, only 31% of learning (sum of fast and slow states) was expressed during acceleration (p = 0.31, Table 1), a value that is remarkably similar to the ratio of acceleration duration to deceleration (0.28). As expected, the fast state had greater sensitivity to error (as compared to the slow state), but also suffered from greater trial-to-trial forgetting. Thus, Model 1 (roughly equivalent to changes in the forward model) could account for the fact that both acceleration and deceleration period commands exhibited spontaneous recovery.

However, Model 1 could not account for one aspect of our data: set-breaks. Fig. 5C shows the effect of set-breaks, pooled across all subjects who experienced horizontal errors from Experiments 1 and 2. Set-breaks resulted in decay, but this decay was only expressed in the deceleration commands, not the acceleration period commands. Deceleration periods commands decayed about 30% when a set break occurred during the perturbation period, and about 20% when a set break occurred during an error-clamp period (Fig. 2D). Thus, Model 1 could not account for the fact that forgetting was specific to the deceleration period (Fig. 5D). This was because a single controller required that both acceleration and deceleration exhibit the same decay due to reliance on the same slow and fast states.

This led us to the approach suggested in Model 2, in which two distinct controllers, each with multiple time scales, undergo adaptation. It is possible that these two putative controllers represent the superior colliculus and the cerebellum, or alternatively, two distinct components of the cerebellum.

**Point 12A.** The third issue is that the authors base much of their judgments of model accuracy on transient changes during set breaks. However, this leads to two potential problems; one is that the authors average set-break behavior for error clamps across experiments 1 and 2, but the expected decay over those set breaks is expected to be in the opposite direction (decay toward baseline in experiment 1, or decay toward the adapted state – spontaneous recovery – during experiment 2). Thus by averaging these two datasets together the authors are likely wiping out any potential decay that might be present (e.g., compare Fig. 4B to Fig. 2A).

Agreed. As you noted, our original analysis possessed an important weakness. While generally set breaks would be expected to produce decay in adaptation, a set break during the error clamp period in the spontaneous recovery paradigm could in fact lead to a recovery. Thus, combining participants across Experiments 1 and 2 could have created the illusion that set breaks do not cause error clamp period decay, because set break decay in Experiment 1 was counterbalanced by recovery in Experiment 2.

To evaluate this possibility, we considered error clamp set break decay in Experiments 1 and 2 separately. To calculate decay, we used an absolute metric (total decay in degrees) and a relative metric (total decay as a percentage). Acceleration commands did not show absolute decay in Experiment 1 (t(23)=-0.78, p=0.44), or Experiment 2 (t(19)=1.29, p=0.21). Similarly, acceleration commands did not show relative decay in Experiment 1 (t(19)=-0.55, p=0.59), or Experiment 2 (t(11)=-0.49, p=0.64). Thus, acceleration period commands did not exhibit set break decay in Experiments 1 and 2. Thus, combining data across Experiments 1 and 2 had no qualitative effect on our initial conclusion that acceleration period commands showed no decay during error clamp period set breaks.

In contrast, deceleration period commands showed absolute decay in Experiment 1 (t(23)=-2.27, p=0.033), but not Experiment 2 (t(19)=-0.41, p=0.688). Similarly, deceleration commands showed relative decay in Experiment 1 (t(19)=-3.65, p=0.002), but not Experiment 2 (t(11)=1.21, p=0.25). Thus, in line with your criticism, combining participants across Experiments 1 and 2 affected our initial conclusion that deceleration commands exhibited no error clamp set break decay. Instead, deceleration commands exhibited error clamp set break decay in Experiment 1, but not Experiment 2, possibly because Experiment 2 used a spontaneous recovery paradigm.

We have now corrected this issue in the revised manuscript (Fig. 5D). Now, when we consider decay due to error clamp period set breaks, we focus solely on Experiment 1, where decay is not confounded by recovery as in Experiment 2. However, we still combine participants from Experiments 1 and 2 when calculating decay during perturbation period set breaks. In Experiment 2, perturbation period set breaks occur prior to the perturbation reversal and thus are analogous to the perturbation period set breaks in Experiment 1. We conclude that set breaks produce decay in deceleration commands during both perturbation and error clamp periods.

Given this important correction, we revisited why our computational models (both Models 1 and 2) suggested that deceleration period decay was specific to perturbation periods, but not error clamp periods, when this was not true in Experiment 1. Below, we show each model fit to behavior in Experiment 1, as reported in Figs. 5A&B in the original manuscript (Fig. R5). Indeed, it does appear that both models struggled to match decay in deceleration commands during the error-clamp period, especially after the set break occurred. For example, we have highlighted the last error clamp decay period with a magenta arrow. Note how each model overpredicted deceleration commands during this period.

**Fig. R5.** Models from the original manuscript, as fit to behavior in Experiment 1. Behavior exhibits savings but the models cannot: both models struggled to match deceleration commands in repeated exposure to the perturbations. Both models had trouble fitting the decay during the error-clamp periods.

To understand why each model overpredicted deceleration commands during error clamp periods, we noted another key limitation in each model. Over time, each model exhibited a worsening ability to track total adaption during deceleration periods. For example, we highlighted the last learning period with a black arrow. Note how each model significantly underpredicted deceleration period commands during this period. These limitations suggested that each model was exhibiting a trade-off between achieving greater decay during error-clamp periods and achieving more complete adaptation during subsequent learning periods. Thus, we reasoned that improving each model’s ability to re-adapt to the perturbation during each learning period, may also improve the model’s ability to capture the total extent of decay during error-clamp periods.

We realized that a primary reason why each model underpredicted re-adaptation to each perturbation exposure, was that it could not exhibit a key hallmark of learning: savings. That is, upon re-exposure to a perturbation, the rate and extent of learning increases. In the state-space model, savings is due to increases in error sensitivity (Herzfeld et al., 2014; Coltman et al., 2019). However, in Models 1 and 2, error sensitivity was constant; thus, these models could not exhibit savings. Therefore, in the revised manuscript, we updated Models 1 and 2 so that they allow error sensitivity to increase linearly with each exposure to the perturbation (see Methods). AIC and BIC confirmed that our revised variable error sensitivity models were more likely to explain the measured data (described in Point 3 in this response to reviewer comments). Also note that these changes to Models 1 and 2 increased the statistical evidence for Model 2 over Model 1 (described in Points 3 in this response to reviewer comments).

As we had speculated, the updated variable error sensitivity models more accurately tracked measured adaptation during perturbation periods, as well as decay during error-clamp periods. For example, note the improvement in model fit during the adaptation and error-clamp periods highlighted with the black and magenta arrows below (the revised model fits are shown in black and the original model fits are shown in the dashed green line).

**Fig. R6**. Revised models fit the data better than the models in the original manuscript. The output of the models in the original manuscript is shown with the green dashed line. Black lines show output of the updated models. Note the better fit to the perturbation periods, and the better fir to the error-clamp periods.

Next, we asked whether adding variable error sensitivity affect the set break decay predicted by each model. We calculated the set break decay predicted by each model in Experiments 1 and 2. Given your comment, we now predict error clamp period set break decay using Experiment 1 only. However, we combine Experiments 1 and 2 to predict perturbation period set break decay.

|  | **Fig. R7**. In the measured data, acceleration period commands exhibited little or no decay in both the perturbation block and the error-clamp block. This was inconsistent with Model 1. |
| --- | --- |

Model 2 better matched the measured data in that it predicted set break decay in deceleration period commands (bottom row of Fig. R7), but not acceleration period commands (top row of Fig. R7). Model 1 however predicted set break decay in both acceleration (top row) and deceleration commands (bottom row), which was inconsistent with the measured data. Critically, note that each model now predicted decay in deceleration commands during error clamp period set breaks. Thus, these changes to Models 1 and 2 allowed the models to capture error clamp period set breaks (bottom-right), but these models still differed in their abilities to account for differential decay in acceleration and deceleration period commands (compare top and bottom).

To summarize, we agree you that to analyze error clamp period set breaks, participants should not be combined across Experiments 1 and 2. In the revised manuscript, we now calculate error clamp period set break decay in Experiment 1. To better capture this decay, we revised Models 1 and 2 to allow error sensitivity to increase with each exposure to the perturbation (Methods). The variable error sensitivity models better described the measured behavior in Experiments 1 and 2. The new predictions of the models are shown in Fig. 5. We have updated our conclusions accordingly to state that set breaks induce deceleration period decay during error clamp periods, in addition to perturbation periods. Importantly, however, only Model 2 could predict separate decay patterns in acceleration and deceleration period commands. That is, Model 1 required identical decay in acceleration period and deceleration period commands, which we did not observed in the data. For this reason, both AIC and BIC indicated that Model 2 was more likely to explain the measured data than Model 1 (Supplemental information, Fig. S2).

**Point 12B.** The other potential issue is that if indeed there is much less adaptation occurring in the acceleration phase, forgetting may be present but so small that it is not reliably detectable relative to the noise in the data (e.g., if forgetting is proportional to the amount of adaptation achieved); this could lead to the erroneous conclusion that there is only decay in the deceleration phase.
Agreed. We performed additional analyses to check for this possibility.

In principle, if acceleration and deceleration commands are related by a scaling factor (as in Model 1), theoretically, noise would not impact our ability to detect a statistically significant change in acceleration commands. That is, because the t-statistic is scale invariant, noise would not weaken statistical power to detect set break decay in acceleration commands had they been a scaled version of deceleration commands. For this reason, we reported decay in the original manuscript as a percentage relative to total adaptation, not the absolute change in adaptation (in degrees). This relative set break decay measure takes into account differences in total adaptation between acceleration and deceleration commands; smaller absolute acceleration decay is amplified by divisively normalizing by a smaller total adaptation extent. The relative decay measure suggested that set break decay was specific to deceleration period commands, not acceleration period commands. These results are visually depicted in the figure below. Set breaks during the perturbation periods led to decay in deceleration commands (-25±3%, t(39)=-7.85, p<0.001) but not acceleration commands (-6±7%, t(39)=-0.90, p=0.37). Similarly, set breaks during the error clamp periods led to decay in deceleration commands (-18±4%, t(19)=-4.73, p<0.001) but not acceleration commands (1±8%, t(19)=0.11, p=0.92).

|  | **Fig. R8**. Percent change in acceleration and deceleration period commands during set breaks. |
| --- | --- |

Next, we conducted additional analyses to test whether set break decay was specific to deceleration commands. First, divisive normalization could have increased variability in our relative set break decay measures, obscuring statistically significant set break changes in acceleration commands. To check this possibility, we calculated an absolute decay measure, where set break decay is not divisively normalized. Total set break changes in acceleration and deceleration period commands are shown in the figure below (Fig R8).

We found that the absolute decay measures agreed with our relative decay measures. Deceleration period commands exhibited decay during perturbation period set breaks (t(43)=-9.48, p<0.001) as well as error clamp period set breaks (t(23)=-2.27, p=0.033). Acceleration period commands did not show statistically significant decay during perturbation period set breaks (t(43)=-1.62, p=0.112) or error clamp period set breaks (t(23)=-0.78, p=0.441). Thus, it did not appear that divisively normalizing set break decay altered our primary conclusion that decay was specific to deceleration period commands.

|  | **Fig. R9**. Absolute change in acceleration and deceleration period commands during set breaks. |
| --- | --- |

Finally, we performed a new analysis that controlled for differences in adaptation extent. On average, acceleration period commands adapted approximately 0.75 degrees. Deceleration commands exceeded this level even prior to the initial perturbation period set break. The set break at which deceleration commands were smallest occurred during the first error clamp period. At this point in Experiment 1, adaptation in deceleration commands was slightly less than 0.8 degrees. In fact, there was no statistically significant difference in deceleration period commands prior to the first error clamp set break, and the acceleration commands achieved prior to perturbation periods in Experiment 1 (t(23)=0.585, p=0.564; compare pre set break bars below). Critically, despite the fact that acceleration and deceleration commands exhibited similar adaptation during these periods, set breaks resulted in decay in deceleration commands (t(23)=2.45, p=0.022; compare pre-set break and post, red, below), but not acceleration commands (t(23)=0.42, p=0.679; compare pre-set break and post, blue, below). Furthermore, note that only deceleration decay was statistically significant, even though its standard deviation was nearly twice the standard deviation in acceleration decay (compare error bars below).

|  | **Fig. R10**. Controlling for differences in adaptation extent. Before the 1^st^ error-camp period, deceleration period commands were approximately equal to the acceleration period command during the perturbation period. This allowed us to control for extent of adaptation. Decay was present in the deceleration period commands but not acceleration. |
| --- | --- |

This comparison provided even stronger evidence that decay was specific to the deceleration period; this remained true even when controlling for the total amount of acceleration and deceleration adaptation, and when noise in deceleration commands was larger than acceleration commands). Thus, we do not feel that noise is altering our conclusion that acceleration period commands did not exhibit decay with time passage; rather, acceleration and deceleration commands exhibited an underlying difference in the susceptibility to the passage of time. This is one critical piece of evidence supporting Model 2 over Model 1; when deceleration period commands can change without any change to acceleration commands, it suggests that these commands are supported by separate controllers.
We now discuss the possibility that noise could have impacted our conclusions concerning set break decay (Supplementary Information, Fig. S4). We again thank the reviewer for noting this concern and feel that the revised manuscript more strongly demonstrates how deceleration commands decay due to time passage, but not acceleration commands.

Additional Comments:

**Point 13.** line 124: Running a MANOVA across so many trial bins is difficult to interpret as there is no sense of exactly which bins are driving the main effect; thus to draw a conclusion about a systematic change over time seems a little inappropriate from such an analysis.
The RMANOVA tests for any significant difference in means due to the experimental factors among the various repeated measures but says nothing about which measure(s) may be different, so this point is well taken. Nevertheless, we chose to use the RMANOVA because of the interaction term. The main affects of trial bin and movement phase indicate that both variables have an affect on adaptation, but crucially, the significant interaction term indicates that the relationship between trial bin and adaptation is different for the 2 movement phases. The later calculation of the learning rates (lines XXX) confirms this.

**Point 14.** lines 151-153: It isn’t clear why these subjects should be excluded. The authors should report how many participants were removed in this manner. However, this method is problematic because it will bias the results toward finding significant differences.
As noted in Point 12B in this document, acceleration and deceleration commands adapted to different extents. Because set break decay is proportional to total adaptation, deceleration commands would be expected to exhibit greater decay. Therefore, to properly compare acceleration and deceleration decay we normalized total decay by the total adaptation achieved prior to the set break. Because this relative metric normalizes to total adaptation, this divisive operation becomes unstable when total adaptation nears zero. Thus, we specified a lower bound on adaptation that was required for each participant to be included in the analysis.

We agree that this method could in principle bias our statistical results towards significance. With that said, our comparison between Models 1 and 2 principally depends on our inability to detect statistical significance in acceleration period set break decay. Thus, excluding participants would more likely bias our result away from this primary conclusion, rather than towards our primary conclusion. In this sense, it more likely adds confidence to our primary acceleration set break decay results.

Nevertheless, in the revised manuscript, we now also report absolute decay, in addition to our relative decay measure. Because this absolute measure does not use divisive normalization, it does not require participant exclusion. As reported in Point 12B, this absolute decay measure agreed with our relative decay measure: deceleration commands exhibited decay during perturbation period set breaks (t(43)=-9.48, p<0.001) as well as error clamp period set breaks (t(23)=-2.27, p=0.033). Acceleration period commands did not show statistically significant decay during perturbation period set breaks (t(43)=-1.62, p=0.112) or error clamp period set breaks (t(23)=-0.78, p=0.441). Thus, it does not appear that excluding participants in our relative decay measure altered our primary conclusion that decay was specific to deceleration period commands. The updated absolute and relative decay measures are reported in the Supplementary Information, Fig. S4.

**Point 15.** line 343: Why did the authors choose to compare trial n and trial n+1, versus using the triplet method that others have used previously (e.g., comparing trial n-1 to trial n+1)? Trial n is potentially contaminated by the surprising target jump (which is fairly obvious in the cross-axis case).
The triplet method is one that we have used for analysis of adaptation in reaching movement. In this method, we begin with an error-clamp trial in trial n-1, then in trial n the movement experiences an error, and then on trial n+1 the movement is again confined to an error-clamp. The difference between behavior in trials n+1 and n-1 is a proxy for learning from error in trial n.

For reaching experiments, errors are experienced during the movement. Thus, the movement in trial n is contaminated by the feedback response to error. However, in saccade experiments, the movement concludes and then the visual system experiences the error. This means that for saccades, trial n can be viewed as a movement that is not contaminated by response to error. Once the saccade in trial n concludes, the error that follows results in adaptation, which then is expressed in the following movement in trial n+1. Thus, in saccade adaptation it is appropriate to measure adaptive response to error in trial n via a comparison of the movement in trial n with trial n+1.

**Point 16A.** line 372-373: Why analyze when the correction moved in the direction of the error, versus the time when the saccade trajectory differed from baseline?
This analysis is measuring response to error in a single trial, and thus compares saccade in trial n with saccade in trial n+1. Comparison to baseline is not appropriate because despite the zero-mean nature of the perturbation, behavior can drift due to local periods in which the mean is not zero. As far as measuring the time when the correction moved in the direction of the error versus when it deviated from the previous trial, that is a reasonable question; however, as you pointed out in Point 16B, the initial deviation of the trajectory is, on average, in the wrong direction, exaggerating the error rather than correcting it. Therefore, it seemed unlikely that this initial deviation was due to the adaptive response to the error.

**Point 16B.** It isn’t entirely clear why a saccade might deviate in the wrong direction early in the movement, but it could be deliberate (e.g., to compensate for ocular dynamics/inertia). In particular, it is interesting to note that this initial motion in the wrong direction does not appear in the first trial of experiments 1 and 2 (fig. 6F), suggesting it may indeed be a response to the fluctuating single-trial condition.

This is indeed an interesting and puzzling aspect of our data. However, it was consistent across all experiments, though most pronounced in Exp. 3 (see Fig. R2D above for single trial data in Exp. 1&2). Although we cannot fully explain it, our data do show an interesting possibility. We found that small changes in the starting position of the saccade from one trial to the next had a measurable effect on saccade trajectory. Surprisingly, this effect was primarily expressed during the acceleration phase of the saccade (Fig. R11). As expected, the change in saccade trajectory was in the opposite direction as the change in the starting position. That is, a saccade that started slightly further to the right tended to have slightly less horizontal displacement than the previous saccade. However, this effect was greater during acceleration than deceleration. This is important because in Experiment 3 there was a correlation between the error experienced on trial n and the change in starting position for trial n+1 (0.012 ± 0.005° following H+, -0.005 ± 0.004° following H-). For this reason, we removed trials with a change in horizontal starting position greater than 0.5° from our analysis of Experiment 3. The effect of the change in starting position was also present during the baseline periods of Experiments 1 & 2 when there were no errors present (Fig. R11C). This observation adds further support to the idea that there may be separate controllers that contribute to acceleration and deceleration periods of a saccade.

We added this result to the supplementary information.

**Fig. R11**. Effect of change in horizontal starting position (CHSP) of the saccade in trial n with respect to the saccade in the previous trial. The y-axis quantifies the trial-to-trial change in saccade trajectory during the acceleration and deceleration phases of the saccade. **A.** Effect of change in horizontal starting position. Data from Exp. 3. Leftmost bin is CHSP < -0.5°, next is -0.5° < CHSP < 0°, third bin is 0° < CHSP < 0.5°, and the rightmost bin is CHSP > 0.5°. **B.** Close up of middle two bins in part A. **C.** Effect of change in starting position in the baseline periods of Experiments 1 & 2. Bins are quintiles of CHSP. As in Experiment 3, there is a strong effect that is mainly expressed during acceleration rather than deceleration.

**Point 17.** line 389: Does Model 2 predict that movement in the acceleration phase should go in the opposite direction in Experiment 3? If not, it seems that neither model is doing a good job of explaining the observed behavior, and there remains no clear winner between the two proposed models.
This is an aspect of our data that remained unexplained by both models. However, the comparison between the two models was based on the empirical data regarding the effects of set breaks: whereas Model 1 required that both the acceleration and deceleration phases show decay, the measured data exhibited little or no decay for the acceleration phase. This was consistent with Model 2. Thus, statistical comparison between the two models provided evidence in support of Model 2.

**Point 18.** section starting 428: This model is reminiscent of the old bang-bang controller models of the cerebellum (in which one side of the cerebellum accelerated the eye and the other side of the cerebellum decelerated it); the authors should review this literature and consider how their model is similar or different to those models. It would also be helpful if the authors could better explain in their model how the same circuit can govern both the acceleration and deceleration phases of a movement depending on the sign of the error and the direction of the primary saccade (e.g., based on lines 445-455, presumably the right fastigial nucleus responding to anti-preferred rightward errors and affecting acceleration would also respond to preferred leftward errors and affect deceleration – how would that work?).

In considering your comments, and reflecting more on the empirical data, we realized that an alternative and potentially simpler way to link the behavioral data to neurophysiology is to suggest that the adaptive changes to the acceleration phase may reflect changes in the way the superior colliculus responds to the visual stimulus, whereas the adaptive changes to the deceleration phase may reflect the way that the cerebellum adds additional commands to the ongoing movement. The two timescales of adaptation in the cerebellum may be associated with a fast timescale in the Purkinje cells, and a slow timescale in the deep cerebellar nucleus.

The conjecture that during the acceleration phase, adaptive changes may be associated with the colliculus is more speculative because current work has not found strong evidence for collicular changes during saccade adaptation (Frens and Van Opstal 1997; Quessy et al. 2010). However, in other adaptation tasks such as reaching in a visuomotor rotation paradigm, there is good evidence that part of the adaptive response is via remapping of the target. Notably, by allowing subjects to have greater preparatory period (reaction time), subjects increase the amount of remapping. This remapping may also be present in saccade adaptation. Future experiments can test for this by varying the reaction time in response to presentation of a stimulus in a cross-axis adaptation protocol.

**Reviewer #3**

The study investigates behavioral signatures of fast and slow processes during saccadic motor learning that go back to the occurrence of spontaneous recovery after extinction. I have reviewed this manuscript before for a different journal and I think that in the present version the authors much better present what their data can actually show. It is a fine-scale examination how fast and slow processes are expressed in the saccadic acceleration and the deceleration phase during learning, set breaks and error-clamp trials. The manuscript now builds up this aim in the introduction and focusses on this strength in the thorough analysis of experimental data.

**Point 19.** I have continuing concerns about the modelling part. From the modelling the authors aims to conclude that learning relies on two adaptive controllers for acceleration and deceleration that are each supported by a slow and a fast process. This is an interesting hypothesis. However, in my understanding, the analysis shows that this hypothesis is not confirmed — model 2 is not superior to the simpler model 1. Still, the authors argue otherwise. As this remains a central point about the conclusions are drawn from the study, I would like to ask for clarification on this point.
Thank you for so carefully reviewing our work. In response to your comments, we made changes to the models. These changes have significantly improved fits of both models, and further distinguished Model 2 as the more likely candidate.

We began by revisiting Models 1 and 2. A closer look revealed two primary issues. In the original manuscript, in Exp. 1 both models exhibited a worsening ability to track adaptation: with each additional perturbation block, actual performance appeared to improve more than what the models could account for. For example, in the figure below we highlight the last perturbation block with a black arrow. Note the under-estimation in each model’s fit to the deceleration period commands. Furthermore, we observed that both models failed to capture the decay during the error clamp periods. For example, we highlight the last error clamp period in the figure below with a red arrow. Note how both models over-estimated deceleration commands during this period.

Fig. R1. Models from the original manuscript, fit to data from Exp. 1. Behavior exhibits savings but the models cannot: both models struggled to match deceleration commands in repeated exposure to the perturbations (black arrow). Both models had trouble fitting the decay during the error-clamp periods (red arrow). Model: black trace.

These two problems are related: each model was exhibiting a trade-off between achieving greater decay during error-clamp periods and achieving more complete adaptation during subsequent learning periods. Thus, we reasoned that improving each model’s ability to re-adapt to the perturbation during each learning period, may also improve the model’s ability to capture the total extent of decay during error-clamp periods.

We realized that a primary reason why both models under-estimated performance in the perturbation block was that the models could not exhibit savings, i.e., the fact that upon re-exposure to a perturbation, the rate and extent of learning increased. In the state-space model, savings is due to increases in error sensitivity (Herzfeld et al., 2014; Coltman et al., 2019). However, in Models 1 and 2, error sensitivity was constant; thus, these models could not exhibit savings.

To remedy the problem, in the revised manuscript we updated Models 1 and 2 so that error sensitivity was able to change with each exposure to the perturbation (Herzfeld et al. 2014). The new variable error sensitivity models solved both problems that the original models had (Fig. R2). For example, note the improvement in model fit during adaptation and error-clamp periods highlighted with the black and magenta arrows (the revised model fits are shown in black and the original model fits are shown in the dashed green line).

**Fig. R2**. Revised models fit the data better than the models in the original manuscript. The output of the models in the original manuscript is shown with the green dashed line. Black lines show output of the updated models. Note the better fit to the perturbation periods, and the better fir to the error-clamp periods.

These variable error sensitivity models each possess two additional parameters than their predecessors. Both AIC and BIC demonstrated that the variable error sensitivity models were more likely to explain the measured data across Experiments 1 and 2 (Fig. R3). That is, in all cases AIC and BIC suggested that more participants were better described by a model where error sensitivity increased over time (positive points in the graph indicate participants that were better fit by a model with a variable error sensitivity; error-bars at right show mean ± SEM across individual BIC differences). For more details on this analysis please see Point 3 above.

**Fig. R3**. Comparison of the current version of the models (variable error sensitivity) with the original version in the initial submission of the manuscript.

Therefore, in the revised manuscript, the constant error sensitivity models were replaced by the variable error sensitivity models. As we had speculated, the updated variable error sensitivity models more accurately tracked measured adaptation during perturbation periods, as well as decay during error-clamp periods.

We next compared Models 1 and 2 using BIC. Reviewer 1 requested that we also compare these models with an additional criterion, because relying on one criterion alone is risky. Therefore, in the revised manuscript, we also compare Models 1 and 2 using AIC. The results of these model comparisons are shown below. In **A** we show the difference in BIC between Models 1 and 2 for each individual participant across Experiments 1 and 2. In **B** we show the same but for AIC.

**Fig. R4**. Comparison of Model 2 with Model 1.

In the original manuscript, BIC indicated that 35 participants were better described by Model 1 (coupled controllers) and 29 participants were better fit by Model 2 (independent controllers). However, now that we have improved both models, these odds have shifted towards Model 2. Now, BIC suggested that 37 participants were better fit by Model 2, whereas 27 participants were better described by Model 1 (an average BIC improvement of 13.7 across individual participants, t(63)=2.34, p=0.023). Summed BIC evidence across all participants favored Model 2 (total BIC difference of 875.5). Notably, AIC model comparison provided even stronger evidence for Model 2. As shown in Fig. R4**B**, AIC suggested that 45 participants were better described by Model 2, whereas 19 participants were better described by Model 1 (an average AIC improvement of 18.4 across individual participants, t(63)=3.14, p=0.003). Summed AIC evidence across all participants favored Model 2 (total AIC difference of 1175).

In summary, we noted that our original models could not exhibit savings, a feature that was present in our data. To solve this limitation, we improved the models by allowing for variability in the error sensitivity parameter using the algorithm suggested in Herzfeld et al. 2014. With this change to Models 1 and 2, both now fit the data better. Importantly, BIC and AIC both suggest that Model 2 is a better fit to the data. We cover these points in more detail in response to Points 20-25 below, and we highlight other features of the analysis not described here. In addition, we also describe some limitations in our modeling that we have tried to better highlight in the revised manuscript.

**Point 20.** Model 1 is the simpler model in which a slow and a fast process are expressed to a different amount in acceleration and deceleration (2 * trial-based retention + 2 * error sensitivity + 2 * time-based retention + 1 * percentage of expression within acceleration/deceleration = 7 parameters). Model 2 should represent the alternative hypothesis - that acceleration and deceleration are each controlled by different fast and slow processes.

We thank the reviewer for noting these aspects of Models 1 and 2 – some details about these models are indeed not apparent in the Results section of the original manuscript. You are indeed correct; Model 1 possessed a total of 7 parameters. Please note that after adding the variable error sensitivity terms to the revised model (see Points 19), 2 additional parameters have been added (1 to allow an increase in slow state error sensitivity, 1 to allow an increase in fast state error sensitivity) bringing the total count to 9 for Model 1 (and 10 for Model 2).

Models 1 and 2 represent alternate hypotheses. Model 1 represents that hypothesis that there is a single adaptive controller that learns from error via its two states, but that only a fraction of that learning can be expressed during the acceleration phase. Thus, in Model 1, acceleration and deceleration period commands are related by a scaling factor, and are co-dependent. This is not the case in Model 2. In Model 2, acceleration and deceleration periods are supported by separate slow and fast states. For this reason, there is no constraint that acceleration commands are related to deceleration commands through a constant scaling factor. Furthermore, in Model 2, acceleration and deceleration commands exhibit independent decay following a set break. More specifically, Model 2 allows the fast deceleration state to completely decay, while completely retaining the fast state for acceleration, after a set break. Thus, Model 2 does not enforce any direct coupling between slow and fast states across the acceleration and deceleration periods.

In the revised manuscript, we describe Models 1 and 2 in greater detail in our Results. We hope that this description gives the reader better intuition about these models. We agree that this intuition is not complete without describing finer details about retention factors, error sensitivities, etc. as described in Point 21 below. Therefore, we have also added clarification to our Results about these parameters, and assumptions that govern Model 2’s exact form.

**Point 21.** Firstly, it important to note that the exact version of model 2 is generated based on a preceding comparison of different model variants in the methods section, examining different combinations of shared parameters across the four states. The final version consists of 2 * trial-based retention (fast and slow, shared across acceleration and deceleration) + 4 * error sensitivity (fast and slow for each acceleration and deceleration) + 2 * time-based retention (a fast deceleration parameter and a shared one for the remaining three combinations) = 8 parameters. This analysis is suitable but please note that the final version of model 2 reflects a complex interplay of different rates in which a clear separation between acceleration and deceleration with each fast and slow is reduced to error sensitivity. Trial-based retention is shared between acceleration and deceleration and time-based retention is special for fast deceleration but the same for the other three states (slow acceleration, fast acceleration and slow deceleration).

Your reasoning is spot on. Model 2 was indeed selected from different model variants, as described in the Methods. In theory Model 2 could have possessed a total of 16 parameters: separate (1) retention factors, (2) error sensitivities, (3) set break decay factors, and (4) rates of error sensitivity increase, for each of the 4 states. To prevent this 16-parameter model from overfitting the data, we conducted a series of model comparisons to reduce the parameter count. We recapitulate these here, as they have changed slightly in the revisions to the current manuscript.

First, as in the original manuscript, BIC model comparison suggested that the slow acceleration and deceleration states, as well as the fast acceleration and deceleration states, had the same retention factors. This likely reflected the observation that acceleration and deceleration commands decayed at the same rate during error clamp periods. Second, similar but slightly different from the original manuscript, model comparison indicated that the best Model 2 variant had two set break decay parameters: one for the slow state of deceleration and one for the fast state of deceleration; the slow state on the other hand, did not exhibit set break decay. This was related to the observation that only deceleration commands decreased after a set break, but that this decrease differed during perturbation periods and error clamp periods (thus requiring a different decay parameter for the slow and fast state of deceleration). Lastly, model comparison indicated that the best model required two parameters to account for increases in error sensitivity: one which applied to how rapidly acceleration (slow and fast) states increased their error sensitivity and one which applied to how rapidly deceleration (slow and fast) states increased their error sensitivity.

We agree that these points about Model 2 provide important insight into this model, and its limitations. We now highlight these points in our Results section when introducing Models 1 and 2.

**Point 22.** The following comparison of model 2 with model 1 reveals that both models fit well to the data. This is strongly confirmed by visual inspection. The only small difference that the authors claim is separating goodness of fit between model 1 and 2 lies in set-breaks during learning (not during learning itself, nor during error-clamp or set-breaks during error-clamp). Hence, this small difference relies on temporal decay — a parameter of model 2 that is not specific to the hypothesis.

We appreciate these critical points. We agree that both models fit well to the data, as is evident by visual inspection. Indeed, the primary difference between these models relates to decay resulting from set breaks. We should note that in response to Reviewer 2 (Point 12A), we recalculated our measures of set break decay in the revised manuscript. Specifically, because set breaks during the error clamp period in Experiment 1 are expected to cause decay, but set breaks during the sole error clamp in Experiment 2 may lead to recovery, we no longer combine Experiments 1 and 2 when calculating how much decay occurs in an error clamp period set break, and instead consider only Experiment 1 (which is not complicated by spontaneous recovery).

The new analysis suggests that set breaks lead to decay during both perturbation periods and error clamp periods, but solely in deceleration period commands. The updated results are shown in the figure below (black, data). Similar to the original manuscript, this deceleration-specific decay can only be accounted for by Model 2. For example, note how Model 1 predicts acceleration period decay during both perturbation and error clamp periods, but Model 2 predicts decay only in deceleration commands.

|  | **Fig. R5**. In the measured data, acceleration period commands exhibited little or no decay in both the perturbation block and the error-clamp block. This was inconsistent with Model 1. |
| --- | --- |

As described in more detail above (Point 12A), our corrected analysis suggests that set breaks produced decay during both perturbation periods and error clamp periods, but only in deceleration period commands. The updated results are shown in the Fig. R5. This deceleration-specific decay can only be accounted for by Model 2. For example, note how Model 1 predicts acceleration period decay during both perturbation and error clamp periods, but Model 2 predicts decay only in deceleration commands.

We feel this is a critical observation that speaks to a fundamental difference between the hypotheses represented by Models 1 and 2. In Model 1, acceleration and deceleration control is tethered. In other words, when acceleration commands increase, so too must deceleration commands and by the same proportion. This model represents the idea that acceleration commands and deceleration commands are derived from the same controller. While Model 1 does produce a reasonable fit to the data, it breaks down in response to a set break. After a set break, only deceleration period commands decay, by about 25%. What this means is that at the very least, 25% of deceleration commands are private to a deceleration-specific control process. Adaptation can be altered by 25% during the deceleration period, without consequence to acceleration period commands. Thus, only a model where at least some part of deceleration control is independent of acceleration control is consistent with the data. While infrequent, the set breaks are critical in setting a lower bound on the amount of deceleration period commands that must be supported by a separate control process. In a larger sense, Model 2 simply serves as a place-holder for the idea that deceleration period commands can be supported at least in part by a separate controller from acceleration period commands. While this may seem to be a small detail, it is significant enough that BIC and AIC measures select Model 2 as the more likely model, though we admit this evidence is moderate. We hope that our revised discussion better describes and qualifies these central ideas.

We also are more direct in describing the limitations in our model. Here we compare two models, one in which control is completely tethered (Model 1) and one in which control is completely separate (Model 2). This need not be the case. In fact, acceleration and deceleration periods could be supported by a framework not considered here, where some states contribute to both acceleration and deceleration periods, but others are specific to one period or another.

Finally, definitive answers to the exact control architecture may be difficult to achieve through modeling alone. For example, if some learning properties are shared by acceleration and deceleration states (consider for example, retention factors), it may seem as if these states are really one and the same (like Model 1), whereas they may in fact be two neural circuits that possess similar properties. These two possibilities would be best teased apart through neural interventions like stimulation or inactivation. We hope that these ideas provide a more nuanced depiction of our results.

**Point 23.** The authors put forward that BIC analysis results are mixed but that the mean over subjects of BIC difference between model 1 and 2 favors model 2, supported by a t-test. Taking BIC differences is rather unusual and Fig. S2A clearly shows that even then, the distribution is highly skewed. As the authors admit, model 1 was superior for 35 subjects and model 2 for 29 subjects, also shown in Fig. S2B on the right side (model recovery analysis based on the data, 55% to 45% for model 1). If I understand it correctly, the model recovery analysis reveals once more that BIC favors model 1 over model 2 if a) fitted to the real data, b) if fitted to simulated data based on model 1 and c) if fitted to simulated data based on model 2 — simply because model 2 is less complex (one parameter less). Isn’t this exactly what BIC should provide, i.e. correction for model complexity? I cannot understand why the authors argue that the analysis reveals a bias of BIC such that it would not be appropriate in means of model comparison (“BIC becomes biased towards the model with fewer parameters.”) This is not a bias but a benefit of BIC and the reason to use it.

Agreed. Before addressing this point, we should first reiterate that these results have changed in the revised manuscript. As described in Point 19, we have updated our models and also updated the comparisons: each model now includes additional terms which allow error sensitivity to increase over time. These changes greatly improved the Model 1 fit and Model 2 fit to the data (see Point 19). We compared these updated fits using both BIC and AIC (see figure below). As Reviewer 1 notes, using one criterion alone could be problematic as BIC tends to select models with lower complexity, and AIC tends to select models with higher complexity.

|  | **Fig. R6**. Comparing Models 1 and 2 with AIC and BIC. |
| --- | --- |

When we compare our updated AIC and BIC measures, we found that statistical evidence at the individual level shifted towards Model 2. Whereas in the original manuscript, 35 participants were best fit by Model 1, and 29 were best fit by Model 2 according to BIC, in the revised manuscript these counts have flipped; 27 participants were best fit by Model 1, but 37 participants were best fit by Model 2. AIC had even greater evidence for Model 2; 19 participants were best fit by Model 1, but 45 participants were best fit by Model 2. Thus, to differing extents, both AIC and BIC in the revised manuscript support Model 2 over Model 1. This agreement offers stronger support towards Model 2 than the original paper.

In the revised manuscript, we also update our model recovery analyses so that they use the variable error sensitivity Model 1 and Model 2 variants. In addition, we provide two separate analyses, one for BIC and one for AIC. We are not sure that we follow the reviewer’s critique of this analysis. We hope we can explain this better. In the original manuscript, BIC suggested that more participants were better described by Model 1. We used the model recovery analysis to determine whether BIC may have been biased towards Model 1 or Model 2. By “bias” here we mean, that even when data are generated by a given model in simulation, BIC selects the other model as the more likely candidate. These biases can result from numerous sources. Because BIC depends on likelihood, model complexity, and the number of data points (samples), these biases can interact with the experimental paradigm. Thus, it is common to test in simulation, whether one can trust some criterion such as AIC or BIC to successfully recover the correct model, or if these metrics are in your specific case biased towards one model (for another recent example, see Forano and Franklin, 2020).

In the original manuscript, we found that BIC was biased towards Model 1. That is, when we simulated data with Model 1, BIC selected Model 1 as the more likely model in almost all cases. But when we simulated data with Model 2, BIC still selected Model 1 as the most likely model. Thus, BIC was returning the incorrect result. The underlying data truly came from Model 2, but BIC was suggesting that the data came from Model 1. Thus, using BIC led to the wrong conclusion. When a selection criterion is biased in this way, one must question whether this measure is a reliable tool in arbitrating between potential models. Therefore, in the original manuscript, we highlighted that while BIC evidence was mixed with respect to Models 1 and 2, it is also the case that BIC was unfairly biased towards Model 1 in simulation. This lowered our trust in the BIC measure.

We have updated these model recovery analyses in the revised manuscript. Now Models 1 and 2 are simulated with a variable error sensitivity. As in the original manuscript, we simulate Experiments 1 and 2 with Models 1 and 2, and ask which model is more likely to explain a given simulated data set according to AIC and BIC. The results are shown below. Inset **A** shows the model recovery analysis with BIC, and inset **A** shows the model recovery analysis with AIC.

|  | **Fig. R7**. Model recovery analysis. |
| --- | --- |

Fortunately, our variable error sensitivity changes to Models 1 and 2 greatly improved the model recovery process. Bias towards Model 1 was much smaller with the BIC metric. When data were simulated with Model 1, BIC still selected Model 1 extremely frequently. When data were simulated with Model 2, BIC selected Model 2 with a 70% probability. Thus, BIC exhibited higher selection errors when data were simulated with Model 2 than Model 1, but it remained more probable to select the correct model using BIC. Results were similar, but perhaps slightly improved with AIC. When data were simulated with Model 1, AIC selected Model 1 with about 80% probability. When data were simulated with Model 2, AIC selected Model 2 also with about 80% probability. Thus, whether data came from Model 1 or Model 2, AIC had approximately a 20% error rate, indicating that it did not exhibit a strong bias towards the selection of either Models 1 or 2.

The resulting analyses suggested that AIC and BIC were both dependable measures that could be used for model comparison, with a slightly greater confidence in AIC than BIC (given a slightly smaller bias). Thus, AIC and BIC agreed that most actual participants were better fit by Model 2. Model recovery analyses suggested that both measures were relatively trustworthy in model selection. Thus, in the revised manuscript we not only have clearer evidence towards Model 2, but also greater confidence in our model comparisons. We note these updated analyses in Supplementary Information section, Fig. S2.

**Point 24.** Experiment 3 is then presented as a further support for the idea of distinct controllers for acceleration and deceleration as supported by model 2. Experimentally, it reveals a bimodal response in which the acceleration phase of the saccade changes in the opposite direction of error while the deceleration phase changes in the direction of error. This result is interesting, indeed. However, to my mind it cannot clarify the lack of evidence from the modelling. The effect in experiment 3 is in learning from error (not in time-based decay) and specifically learning from single error which is shown to be different from learning of persistent errors (experiment 1-2 with modelling).

The key result of this analysis is that the adaptive response to a single error is present only in the deceleration period. According to Model 1, we would have predicted learning would have produced an adaptive response in the entire trajectory, with perhaps a smaller amount present in the acceleration period. In contrast, the data presents a picture in which the adaptive response is present only in the deceleration period.

**Point 25.** Please correct me if I got anything wrong. I put forward these points because I think we should really be careful with the conclusions that we draw from modelling. I find the data of the present experiment worthwhile to report and I also think that the separate controller hypothesis is interesting and worth testing. If the outcome does not provide evidence in favor of it this should simply be state accurately.

We hope that the changes that we have made to the models, and the new results that we arrived at in comparing the models, have resolved some of the issues and improved the manuscript.
