# Supplementary material for: Adaptive control of movement deceleration during saccades
Source: PLoS Comput Biol. 2021 Jul 6;17(7):e1009176. doi: 10.1371/journal.pcbi.1009176 (PMC8284628; doi:10.1371/journal.pcbi.1009176)
Supplement: Attachment — Submitted filename: Response to reviewers 2.docx [file pcbi.1009176.s009.docx]

The major finding of our study was that the surprising finding that after experience of a single error, the adaptive response in the subsequent trial was limited to the deceleration period. After repeated exposure to the same error, the acceleration period commands also adapted, and exhibited resistance to forgetting during set-breaks. In contrast, the deceleration period commands adapted more rapidly, but suffered from poor retention during these same breaks. Thus, it appeared that adaptive control of saccades benefited from two potentially different systems, one that learned slowly and contributed primarily via acceleration period commands, and another that learned rapidly but contributed primarily via deceleration period commands. Overall, our results suggest that distinct adaptive controllers may contribute to the acceleration and deceleration phases of a single saccade.

In this revision, we made a significant change to our modeling approach. We realized that there was a simpler model that could account for much of our data. This simplified model (Model 2) has the same number of parameters as Model 1, but accounts for the major findings in our data: spontaneous recovery of the acceleration period and deceleration period commands, set-break decay in the deceleration period commands but much smaller decay in the acceleration period commands.

**Reviewer #1:**

The authors revised the paper following my suggestion enough. I recommend this paper to get accepted.

We thank the reviewer for their time and effort in reviewing our paper and for helpful comments on previous versions of it.

**Reviewer #2:**

The authors have answered the majority of my concerns in great detail and in a satisfactory manner. However, two concerns remain.

First, the authors noted in their response to point 11 that there is an untested possibility related to my initial concern, largely that the same behavior could have arisen by a simpler alternative model in which the controller and the forward model partially adapt (particularly if they adapt to different extents). In theory this could result in different degrees of expressed adaptation during the acceleration and deceleration phases, since the controller primarily dominates the response during the acceleration phase while the forward model comes online to steer corrections later in the movement. If the two systems partially adapt to different extents, and if one exhibits temporal decay while the other only exhibits decay in response to trials (e.g., the work of Smith and colleagues), this could arguably explain the results without having to resort to a more complex model with separate two-state controllers for the acceleration and deceleration phases. It would be helpful if the authors considered this simpler alternative. Indeed, the authors seem to discourage this possibility by arguing that the superior colliculus doesn’t show changes in activity; however they then contradict themselves by proposing that the colliculus could be involved in remapping (point 18).

Agreed. In this revision we considered an alternate model and found it to be superior to our previous models. This new model has the same number of parameters as Model 1, and thus has the same complexity, yet can account for the major components of the data that were unaccounted for in that model.

In the current manuscript, Model 2 represents the idea that a system learns to aim the saccade, and another system independently learns to modify acceleration and deceleration commands. This model is inspired by the possibility that some of the adaptation is via the superior colliculus, while some is via the cerebellum. In revised Model 2, there is a single “aiming” state that contributes to acceleration period and deceleration period commands. In addition, the acceleration and deceleration periods commands each possess a separate, independent adaptive state. These three states adapt according to the state-space model:

$$x_{aim}^{(n+1)}=a_{aim}x_{aim}^{(n)}+b_{aim}e^{(n)}$$

$$x_{acc}^{(n+1)}=a_{acc}x_{acc}^{(n)}+b_{acc}e^{(n)}$$

$$x_{dec}^{(n+1)}=a_{dec}x_{dec}^{(n)}+b_{dec}e^{(n)}$$

As a result, the acceleration and deceleration period commands are a mixture of the aimed component and the additions of the acceleration and decelerations states. As in Model 1, a proportion *p* of the shared “aiming” state is expressed during acceleration (and 1 – *p*, expressed during deceleration):

$$y_{acc}^{(n)}=px_{aim}^{(n)}+x_{acc}^{(n)}$$

$$y_{dec}^{(n)}=(1-p)x_{aim}^{(n)}+x_{dec}^{(n)}$$

We found that new Model 2 could reproduce the critical features of the data: spontaneous recovery of the acceleration period and deceleration period commands, set-break decay in the deceleration period commands but much smaller decay in the acceleration period commands. As such, this revised 3-state Model 2 outperformed the previous 4-state Model 2: the revised model had a lower BIC and AIC for 54 and 42 out of 64 participants, respectively. Thus, we used this revised Model 2 in our revised paper.

In the Discussion, we noted the likelihood that both the controller and the forward model might adapt, with the forward model adapting more rapidly. We refer to this via the possibility that the superior colliculus and the brainstem structures represent the optimal controller, while the state estimator is represented by the cerebellum. The possibility of target remapping, representing a change in the optimal controller, was studied by Quessy et al. (2010) and Frens and Van Opstal (1997). They recorded from the superior colliculus and found that during saccade adaptation, locus of activity on the superior colliculus was unchanged, thus making remapping unlikely. However, this is not a universal finding, as an examination of this same question by Takeichi et al. (2007) found some evidence for remapping of the target at the level of the superior colliculus. Overall, currently the latest published work on this topic (Quessy et al. 2010) reported no evidence of change to the controller during saccade adaptation.

Of course, as you note below, lack of evidence for a possibility does not make that possibility unlikely. Indeed, we think it is entirely possible that a component of the adapted response reflects a re-aiming process, that is, some part of the brain learns to aim the saccade to a new target location (as in the revised Model 2). In our revised Discussion, we present this possibility and present an experiment that can go after this question. Using ideas from the reach adaptation literature, re-aiming is much more difficult when there are strict time constraints on reaction time. On the other hand, re-aiming is much easier when reaction times are allowed to become longer. Thus, perhaps one way to test whether there is a component of re-aiming associated with cross-axis saccade adaptation is to limit reaction time. The prediction is that saccades that are generated with short reaction times will exhibit smaller adaptation.

As an aside, it is also important to note that the colliulus represents saccades in 2D but saccade motor commands are subsequently modified (e.g., to reflect the torsion component to obey Listing's law) before the commands are sent to the burst generators; thus there is yet another stage after the superior colliculus at which the motor command may differ between adapted and non-adapted saccades.

Agreed, and thank you for pointing this out. We added this possibility in our Discussion.

The other potential area of concern is that the authors primarily depend on interpretation of a null finding (i.e., lack of a significant change in the acceleration command) to disentangle their models. I would caution the authors about over-emphasizing the lack of observing a significant change as being evidence in favor of no change, and particularly in relying on this finding as the primary means of differentiating between models. It would be helpful if the authors consider how they phrase their findings and their model comparisons in light of this concern, and they should acknowledge in the manuscript that they are relying on interpretation of a null finding as evidence in favor of a lack of a change, and in light of that need to be cautious of the conclusions drawn.

Agreed. Concerns about basing interpretations on a null finding are well taken. There are two important null findings in the paper. First, the absence of significant adaptation in the acceleration phase following the experience of a single error, despite robust adaptation present in the deceleration phase. Second, the absence of significant set break decay in the acceleration phase, despite robust set break decay in the deceleration phase (during both the perturbation and error-clamp periods). However, it is important to note that both these null findings were robust across different experiments that included 100+ subjects (single trial learning null result) and several analysis techniques (set break decay null result).

Specifically, our single trial learning analysis suggested an absence of significant adaptation in the acceleration phase following a single error across Experiments 1, 2, and 3 which included 100+ subjects. We now illustrate this finding in Fig. 5F (data from Experiment 1 and 2), and Fig. 5C (data from Experiment 3). In addition, the control data for Experiment 1 (Figure 2) show a decreased contribution during the acceleration phase (compared to an increased contribution during the deceleration phase) of adapted saccades compared to oblique saccades made to the same endpoint during the baseline period, which is consistent with the idea of separate controllers for acceleration and deceleration.

Secondly, multiple set break decay analyses suggested that little to no decay occurred in the acceleration period commands. This occurred when we used normalized decay measures (Fig. 1), non-normalized decay measures (Fig. S2B), and when controlling for acceleration and deceleration command magnitudes (Fig. S2C).

Nevertheless, we have added passages in our Results section to ensure that these null results are noted, and interpreted cautiously.

**Reviewer #3:**

I thank the authors for their detailed responses and improvement of the manuscript. In response to the criticism that the BIC analysis argued for model 1 instead of model 2, the authors added two learning parameters for error sensitivity to both models. Hence, error sensitivity can increase over time. Firstly, it improved the fit of both models to the data and, secondly, it shifted model superiority according to BIC from model 1 to model 2 (BIC: 37 vs. 27 subjects in favor of model 2; AIC: 45 vs. 19 subjects in favor of model 2). I much appreciate this improvement, the effort taken for the manuscript revision, and the contribution that the data can provide, i.e. a fine-scale examination of how fast and slow processes are expressed throughout the acceleration and deceleration phase of the saccade.

Thank you kindly. We agree that addition of the error sensitivity parameter greatly improved the ability of the various models to account for the data. Indeed, BIC analysis now clearly favors model 2.

However, I must admit that the authors could not rule out my concern about the validity of model 2 with respect to the storyline of the manuscript. The story that the authors make is that learning and spontaneous recovery rely on two adaptive controllers, one for acceleration and one for deceleration, that each rely on a fast and a slow process. Hence, they suggest a four-state framework as an extension of the well-known two-state framework of a fast and a slow state of learning. My doubts in the validity of model 2 for the four-state framework relied on two aspects that still persist: 1) With the aim of parameter reduction, model 2 is generated based on a preceding comparison of different model variants. In the revised version, the final version of model 2 is still a complex interplay of different rates that are shared across acceleration and deceleration or fast and slow states in the following form: 2 trial-based retention rates (fast and slow; hence shared between acceleration and deceleration), 4 error sensitivity parameters (the only parameter with differentiation for all 4 states), 2 parameters for error sensitivity increase (one for acceleration, one for deceleration; hence shared between fast and slow) and 2 time-based retention rates (fast deceleration and slow deceleration; fast and slow acceleration are fixed to 1 such that they do not decay and do not need a parameter). Thus, learning and decay of fast and slow processes spread in a complex way across different phases of the saccade. However, I cannot conclude a clear separation between an acceleration and a deceleration controller, each relying on a fast and a slow state. Firstly, because the parameter reduction of model 2 does not support the hypothesis of a clear four-state framework…

While we agree that the parameter reduction of Model 2 does not show a clear separation into 4 states, we think there are two basic aspects of the data that point toward some degree of independence between the acceleration and deceleration period commands. First, in Exp. 1 and Exp. 2 we observed that set breaks produced forgetting in the deceleration period commands but not acceleration. Second, in Exp. 3 we observed that learning from error produced different patterns of learning in the acceleration and deceleration periods: only the deceleration period exhibited a correction. We were able to confirm this observation by re-analysis of data in Exp. 1 and 2. Thus, these components of the experimental results are inconsistent with Model 1, but can be accounted for by Model 2.

In response to both your concern and a related concern noted by R2, we considered an alternate version of Model 2 in the revised manuscript. This new model has the same number of parameters as Model 1, and thus has the same complexity, yet can account for the major components of the data that were unaccounted for by Model 1.

In the revised manuscript, Model 2 represents the idea that a system learns to aim the saccade, and another system independently learns to modify acceleration and deceleration commands. This model is inspired by the possibility that some of the adaptation is via the superior colliculus, while some is via the cerebellum. In revised Model 2, there is a single “aiming” state that contributes to acceleration period and deceleration period commands. In addition, the acceleration and deceleration periods commands each possess a separate, independent adaptive state. These three states adapt according to the state-space model:

$$x_{aim}^{(n+1)}=a_{aim}x_{aim}^{(n)}+b_{aim}e^{(n)}$$

$$x_{acc}^{(n+1)}=a_{acc}x_{acc}^{(n)}+b_{acc}e^{(n)}$$

$$x_{dec}^{(n+1)}=a_{dec}x_{dec}^{(n)}+b_{dec}e^{(n)}$$

As a result, the acceleration and deceleration period commands are a mixture of the aimed component and the additions of the acceleration and deceleration states. As in Model 1, a proportion *p* of the shared “aiming” state is expressed during acceleration (and 1 – *p*, expressed during deceleration):

$$y_{acc}^{(n)}=px_{aim}^{(n)}+x_{acc}^{(n)}$$

$$y_{dec}^{(n)}=(1-p)x_{aim}^{(n)}+x_{dec}^{(n)}$$

We found that new Model 2 could reproduce the critical features of the data: spontaneous recovery of the acceleration period and deceleration period commands, set-break decay in the deceleration period commands but much smaller decay in the acceleration period commands.

The revised Model 2 has several advantages over our previous Model 2. First, the revised Model 2 relies on 3 adaptive states as opposed to 4. Second, the full parameter set for Model 2 is now 13 parameters (as opposed to 16 parameters initially). Third, it attributes adaptation to separate processes: re-aiming and forward model adaptation.

As in past versions of the manuscript, we tested several revised Model 2 variants to reduce parameter complexity via BIC and AIC-based model comparisons. These comparisons suggested that the aiming state and acceleration state did not possess a set break decay component. Second, they suggested that the rate parameter which determines how fast error sensitivity increases from its initial value, was the same across all 3 states. Critically however, unlike past versions of the manuscript, these comparisons suggested that all 3 states had distinct error sensitivity (*i.e. b*) and retention (*i.e.*, *a*). Thus, in contrast to the past 4-state Model 2, the revised 3-state Model 2 had distinct learning and decay parameters across all 3 states, and by extension, the acceleration and deceleration periods. This revised Model 2 therefore provided a much clearer delineation between all 3 putative adaptive processes.

Overall, the optimal revised Model 2 had 9 total parameters: 3 retention factors for the 3 states, 3 error sensitivities for the 3 states, a decay parameter for the deceleration-only state, an error sensitivity increase parameter, and the *p* parameter which determines how aiming is distributed across acceleration and deceleration (like Model 1). The revised 3-state Model 2 outperformed the previous 4-state Model 2: the revised model had a lower BIC and AIC for 54 and 42 out of 64 participants, respectively. Thus, we used this revised Model 2 in our revised paper.

Finally, because the revised Model 2 possessed 9 parameters, as did Model 1, we no longer required AIC and BIC in their statistical comparison (Fig. S5). Instead, we directly compared log-likelihoods (and did the same in our model recovery analysis). Maximum likelihood estimation suggested that the revised Model 2 better described 47 participants, whereas Model 1 better described only 17 participants.

We hope that these revisions to Model 2 help to address the reviewer’s concerns.

Secondly, because the separation of the saccade into the acceleration and deceleration phases has not been tested against other separations of the saccade.

Our rationale for separating the saccade into acceleration and deceleration is as follows. In our earlier work (Chen-Harris et al. 2008), we reported that adaptation in response to cross-axis endpoint errors produced saccade trajectories that exhibited interesting properties: the motor commands that initiated the saccade changed slowly in the direction that would reduce the endpoint error, but late in the same saccade, the motor commands appeared to steer the eyes further. Our analysis in Chen-Harris et al. (2008) relied on division of the saccade trajectory (i.e., its path) into 4 equal length segments. The segments were defined by dividing the length of the saccade into 4 parts along its primary axis (thus, resulting in 4 chords), as shown in Fig. R1A below. In control trials (before adaptation), we measured the slope of each chord in response to targets at (+15,0), (+15,+1), (+15,+2), and (+15,+3) degrees. We measured the chord slopes in these control trials, as shown in Fig. R1B. We then performed the adaptation experiment and presented the target at (+15,0) and again measured the chord slopes. The results are shown in Fig. R1C below.

**Fig. R1.** Analysis of saccade path during cross-axis adaptation. Saccades were made to horizontal targets at 15^o^. To induce adaptation, the target was jumped by 5^o^ along the vertical direction. (A) Each saccade was divided into 4 segments, called chords, based on the horizontal position. Slope of each segment was measured and labeled as S1, S2, S3, or S4. (B) Control trials in which subjects made oblique saccades to targets at 1, 2, or 3 deg vertical amplitude and 15 deg horizontal amplitude. (C) Data during early, middle, or late trials of adaptation. Note that chord slopes within a saccade increase nonlinearly, indicating presence of curvature. Data from Chen-Harris (2008).

We found that with adaptation, the chord slopes exhibited a remarkable pattern: while the slope gradually increased as the movement progressed toward its endpoint, the rate of change was not linear. Rather, whereas chords 1 and 2 had roughly equal slopes, chord 4 had twice the slope of chords 1 and 2. This illustrated that for the adapted saccades, most of the change was due to commands that arrived late in the movement. Notably, a critical time point during a saccade was roughly at the end of chord 2, that is, midway into the movement. This point along the saccade’s trajectory corresponds approximately to peak speed of the saccade. Thus, empirical evidence from Chen-Harris et al. (2008) provided us with a rationale to divide the saccade based on timing of peak speed, leading to acceleration and deceleration phases.

However, as you noted, by itself this is not a strong piece of evidence. Thus, part of the impetus for Exp. 3 was to ask whether there was independent evidence for the idea that control of the deceleration period is specifically under an adaptive process that is different than the deceleration period. In Exp. 3, we focused on single trial learning, thus allowing us to measure trial-to-trial change in saccade trajectory in response to a single endpoint error (Fig. R2A). We found that following experience of a single error, on the next trial the motor commands changed. We quantified this change via the horizontal and vertical components of the saccade velocity and found that horizontal velocity (Hdot) showed a bimodal response that crossed zero almost precisely at saccade peak speed (Fig. R2B). That is, the motor commands that steered the eyes in the correct direction did not arrive until the deceleration period of the saccade. These results indicated that on average (i.e., across the population), onset of saccade deceleration was a critical timepoint for analysis of Hdot.

**Fig. R2**. Following experience of a single error, correction on the subsequent saccade was primarily during the deceleration period. **(A)** Subjects were presented with a vertical target at 15 deg and occasionally experienced a horizontal error of +5 or -5 degrees. **(B)** Trial-to-trial change in horizontal velocity following experience of a positive or negative endpoint error. Note that the response as measured via Hdot is bimodal, crossing the zero axis almost precisely at onset of deceleration. **(C)** Onset time of correction in the horizontal velocity following experience of a horizontal endpoint error. Mean onset time is about 3 ms following start of the deceleration period. **(D)** Data from Experiments 1 & 2, measuring the response to error following experience of error on the very first trial (velocity on trial 2 minus trial 1, in the direction of the perturbation). The component of Hdot that corrects for the error experienced in the previous trial is entirely in the saccade deceleration period.

Next, we searched for the time during a saccade’s trajectory in which Hdot indicated onset of the corrective command. That analysis showed that the onset of Hdot was statistically indistinguishable from time of saccade peak speed (Fig. R2C). To confirm this finding, we considered the data across all our 100+ subjects and measured how the saccade velocity changed from one trial to the next in response to a single experience of an endpoint error. The results clearly illustrated that the adapted response was present only in the deceleration period of the saccade (Fig. R2D).

In summary, our experiments in Chen-Harris et al. (2008) demonstrated that while adapted motor commands were present during the entire trajectory of the saccade, they were not expressed equally during the trajectory: a nonlinearity appeared to take place at midpoint along the saccade path, which roughly corresponds to saccade peak speed. This empirical evidence provided us with a reason to analyze data based on division of the movement into acceleration and deceleration periods. However, to independently test the idea, we performed Exp. 3 and found that the adaptive response to a single error occurred only during the deceleration period. These empirical lines of evidence compelled us to analyze the data using the time point when the peak speed occurred. These ideas are now highlighted in the both the Results and the Discussion sections.

Thirdly, parameter reduction across fast and slow states was not tested for model 1.
Model 1 included 9 parameters: {*a_s_*, *a_f_*, *b_s,0_*, *b_f,0_*, *d_s_*, *d_f_*_­_, *p,* β*_s_*, β*_f_*}. Our rational for these specific parameters was as follows. The experimental data exhibited spontaneous recovery, which requires inclusion of fast and slow states. The experimental data exhibited differential decay of acceleration and deceleration components due to set breaks, which requires inclusion of two decay parameters *d*. Finally, the experimental data exhibited savings, which requires inclusion of error sensitivity terms. The only possibility is that the two error sensitivity terms are redundant. However, the data in Table 1 demonstrate that β*_s_* is more than an order of magnitude smaller than β*_f_* , with 95% confidence intervals that do not overlap.

2) The effect that separates model 1 and 2 (that both fit well according to visual inspection) relies on set-breaks only, and hence, exclusively on time-based decay in the models. This parameter is not differentiated across all 4 states in model 2, and is hence not specific to the hypothesis. Moreover, the effect is not present during spontaneous recovery. Of course, a separation into fast and slow states is a current and well-known explanation of spontaneous recovery. However, the additional differentiation that the authors make concerning the acceleration and deceleration of the saccade refers to time-based decay during set-breaks, not the phenomenon of spontaneous recovery. I fully support the result that during set-breaks, decay is more present in the deceleration than in the acceleration phase of the saccade, as shown by the experimental data and the models. However, from this interesting detail about temporal decay when no saccades are made, I cannot conclude a general four-state framework of learning and spontaneous recovery.

Agreed. We simplified Model 2 to one that had the same number of parameters as Model 1. In addition, results of Exp. 3 revealed that the adaptive response to the error was present during deceleration, but missing from the acceleration period. We then checked to see if this was also true in Exp. 1 and 2. Results showed that the same pattern was also present in Exp. 1 and 2.

Finally, to quantify the exact time when the commands correctly changed eye trajectory in Exp. 3, we analyzed data of each participant separately and looked for the onset time in which ΔH ̇ corrected the eye trajectory in the direction of the previous error. The results revealed that error compensation did not begin at saccade initiation (Fig. 5E, left subplot, mean 24.14 ± 1.12ms, t(36) = 21.54, p = 7.38X10-22). Rather, the compensation started 3.67 ± 1.05ms following peak velocity, i.e., almost precisely at the onset of deceleration (right subplot, Fig. 5E). This result is interesting because it illustrates an independent confirmation that peak velocity is a critical time point during the saccade trajectory: the response to error that took place in the previous trial is not expressed during the acceleration period, but only after the onset of the deceleration period.

Lastly, it should also be noted that while Models 1 and 2 are best differentiated based on their behavior across set breaks, the revised Model 2 possess error sensitivities and retention factors that are distinct across the 3 adaptive processes: aiming, acceleration, and deceleration.

Nevertheless, we have now added a statement in our Discussion which states a limitation of our work is that the delineation between Models 1 and 2 is primarily based on their time-decay properties.

I do not want to stand in the way of publishing a study that took so much effort, and I really appreciate the work that was done for this study. But I feel that these issues at least need to be discussed and clearly acknowledged in the manuscript if the authors want to insist on their storyline.

We very much appreciate the time you took to review this work. Your comments have certainly made our work better.
